# Elevated mitochondrial membrane potential is a therapeutic vulnerability in *Dnmt3a*-mutant clonal hematopoiesis

**DOI:** 10.1038/s41467-025-57238-2

**Published:** 2025-04-16

**Authors:** Kira A. Young, Mohsen Hosseini, Jayna J. Mistry, Claudia Morganti, Taylor S. Mills, Xiurong Cai, Brandon T. James, Griffin J. Nye, Natalie R. Fournier, Veronique Voisin, Ali Chegini, Aaron D. Schimmer, Gary D. Bader, Grace Egan, Marc R. Mansour, Grant A. Challen, Eric M. Pietras, Kelsey H. Fisher-Wellman, Keisuke Ito, Steven M. Chan, Jennifer J. Trowbridge

**Affiliations:** 1 The Jackson Laboratory, Bar Harbor, ME USA; 2 Princess Margaret Cancer Centre, University Health Network, Toronto, ON Canada; 3 Ruth L. and David S. Gottesman Institute for Stem Cell and Regenerative Medicine Research, Departments of Cell Biology, Oncology and Medicine, Montefiore Einstein Cancer Center, Albert Einstein College of Medicine, Bronx, NY USA; 4 Division of Hematology, Department of Immunology and Microbiology, Anschutz Medical Campus, University of Colorado, Aurora, CO USA; 5 Donnelly Centre for Cellular and Biomolecular Research, Toronto, ON Canada; 6 Department of Medical Biophysics, University of Toronto, Toronto, ON Canada; 7 Division of Hematology/Oncology, The Hospital for Sick Children, Toronto, ON Canada; 8 UCL Cancer Institute, Department of Developmental Biology and Cancer, UCL Great Ormond Street Institute of Child Health, London, UK; 9 Division of Oncology, Department of Medicine, Washington University School of Medicine, St. Louis, MO USA; 10 East Carolina University, Brody School of Medicine, East Carolina Diabetes and Obesity Institute, Department of Physiology, Greenville, NC USA; 11 UNC Lineberger Comprehensive Cancer Center, University of North Carolina at Chapel Hill School of Medicine, Chapel Hill, NC USA

**Keywords:** Ageing, Haematopoietic stem cells, Mitochondria

## Abstract

The competitive advantage of mutant hematopoietic stem and progenitor cells (HSPCs) underlies clonal hematopoiesis (CH). Drivers of CH include aging and inflammation; however, how CH-mutant cells gain a selective advantage in these contexts is an unresolved question. Using a murine model of CH (*Dnmt3a*^R878H/+^), we discover that mutant HSPCs sustain elevated mitochondrial respiration which is associated with their resistance to aging-related changes in the bone marrow microenvironment. Mutant HSPCs have DNA hypomethylation and increased expression of oxidative phosphorylation gene signatures, increased functional oxidative phosphorylation capacity, high mitochondrial membrane potential (Δψm), and greater dependence on mitochondrial respiration compared to wild-type HSPCs. Exploiting the elevated Δψm of mutant HSPCs, long-chain alkyl-TPP molecules (MitoQ, d-TPP) selectively accumulate in the mitochondria and cause reduced mitochondrial respiration, mitochondrial-driven apoptosis and ablate the competitive advantage of HSPCs ex vivo and in vivo in aged recipient mice. Further, MitoQ targets elevated mitochondrial respiration and the selective advantage of human *DNMT3A*-knockdown HSPCs, supporting species conservation. These data suggest that mitochondrial activity is a targetable mechanism by which CH-mutant HSPCs gain a selective advantage over wild-type HSPCs.

## Abstract (web summary)

Clonal hematopoiesis is driven by the selective advantage of mutant hematopoietic stem cells. Here, authors discover that elevated mitochondrial activity is a targetable mechanism by which mutant hematopoietic stem cells gain a selective advantage.

## Introduction

Throughout life, hematopoietic stem and progenitor cells (HSPCs) are vital to sustaining hematopoiesis and maintaining balanced production of mature hematopoietic cells. Aging is associated with the development of clonal hematopoiesis (CH), a condition that occurs when a subset of HSPCs gain a selective fitness advantage based on accrual of somatic mutation(s). The gene most frequently observed to accrue somatic mutations that drive human CH is the DNA methyltransferase *DNMT3A*^1–3^. While CH itself is not a pathological condition, a greater burden of larger clones is associated with increased risk of numerous aging-associated pathologies, including cardiovascular disease^4^, hematologic malignancies^2^, and inflammatory bone loss^5^. Understanding how and why HSPCs with *DNMT3A* and other somatic mutations gain a selective advantage in the context of aging provides an opportunity for intervention to prevent the expansion of clones that increase disease risk.

Given the positive association between aging and inflammation, much work has focused on acute and chronic inflammation as selective pressures promoting clonal selection and CH^6^. Inflammation is proposed to underly the positive associations between CH and smoking, chronic liver disease, autoimmune conditions, increased body mass index, and bone loss^6–9^. While acute and chronic inflammatory states irreversibly deplete wild-type HSCs^10,11^, *Dnmt3a-*mutant and other CH-mutant HSCs (ex. *Tet2, Asxl1*) either resist the response to inflammatory factors or mount a distinct response to inflammatory factors resulting in maintenance of their self-renewal capacity^12–16^. We recently reported that *Dnmt3a*-mutant HSCs have an increased competitive advantage over wild-type HSCs in middle-aged recipient mice^14^ in which we have not observed strong signatures of elevated inflammation^17^. This suggests that other mechanisms contribute to the competitive advantage of *Dnmt3a*-mutant HSCs. Here, we investigated the mechanisms underlying HSPC competition that adapt *Dnmt3a*-mutant HSPCs to aging environments.

## Results

### *Dnmt3a-*mutant HSPCs have a competitive advantage over wild-type HSPCs that is associated with higher mitochondrial respiration

**Fig. 1:**
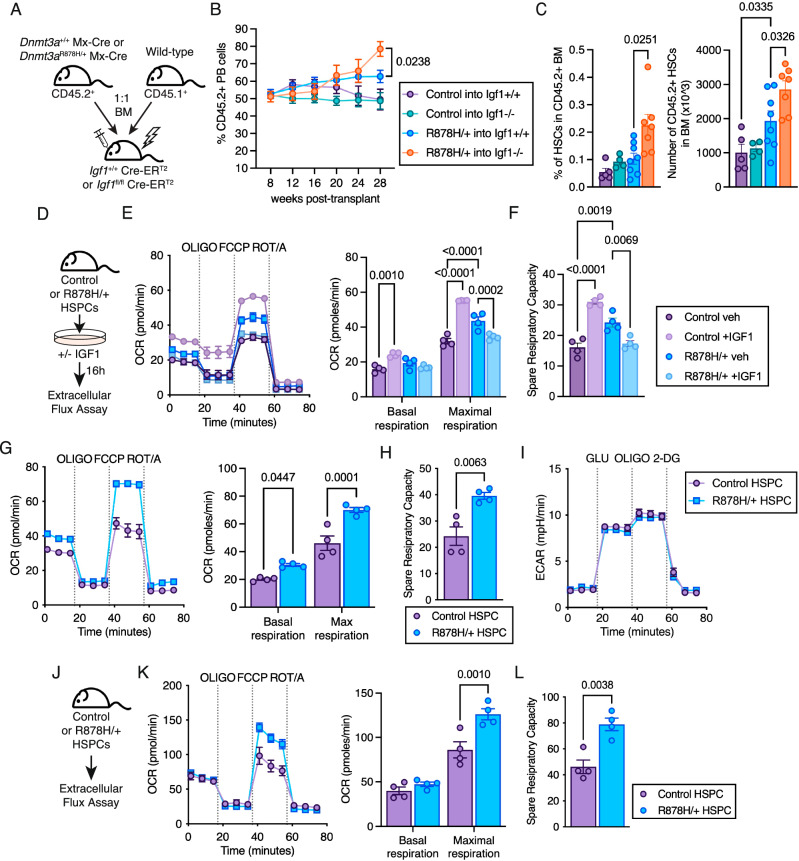
*Dnmt3a*^R878H/+^ enhances oxidative phosphorylation in HSPCs. **A** Experimental design. **B**, **C** Frequency of donor-derived (CD45.2^+^) cells in peripheral blood (**B**), frequency of hematopoietic stem cells (HSCs) in CD45.2^+^ bone marrow at 28 weeks post-transplant (**c**, left) and total number of CD45.2^+^ HSCs in BM (**C**, right) of recipient mice. Symbols represent mean ± SEM, bars represent mean ± SEM with points from biological replicate mice (*n* = 5 control into Igf1 + /+, *n* = 4 control into Igf1−/−, *n* = 8 R878H/+ into Igf1 + /+, *n* = 7 R878H/+ into Igf1−/−). Statistical analyses used two-way ANOVA with Tukey’s multiple comparisons test (**B**) or Brown-Forsythe and Welch one-way ANOVA (**C**). **D** Experimental design. **e** Oxygen consumption rate plot (left) used to quantify basal and maximal respiration (right) and (**F**), spare respiratory capacity in control and *Dnmt3a*^R878H/+^ HSPCs with and without IGF1. **E**, **F** Symbols represent mean ± SEM, bars represent mean ± SEM with points from biological replicate mice (*n* = 4). Statistical analyses used two-way ANOVA with Sidak’s multiple comparisons test (**E**) or one-way ANOVA with Tukey’s multiple comparisons test (**F**). **G** OCR plot (left) used to quantify basal and maximal respiration (right) and (**H**), spare respiratory capacity in control and *Dnmt3a*^R878H/+^ HSPCs after 16 h in vitro culture. **G**, **H** Symbols represent mean ± SEM, bars represent mean ± SEM with points from biological replicate mice (*n* = 4). Statistical analyses used two-way ANOVA with Sidak’s multiple comparisons test (**G**) or one-way ANOVA with Tukey’s multiple comparisons test (**H**). **I** Extracellular acidification rate plot of control and *Dnmt3a*^R878H/+^ HSPCs after 16 h in vitro culture. Symbols represent mean ± SEM from biological replicate mice (*n* = 3). **J** Experimental design. **K** OCR profile plot (left) used to quantify basal and maximal respiration (right) and (**l**), spare respiratory capacity in de novo control and *Dnmt3a*^R878H/+^ HSPCs. **K**, **L** Symbols represent mean ± SEM, bars represent mean ± SEM with points from biological replicate mice (*n* = 4). Statistical analyses used two-way ANOVA with Sidak’s multiple comparisons test (**K**) and unpaired two-tailed *t* test with Welch’s correction (**l**). Source data are provided as a Source Data file.

To narrow down potential mechanisms by which *Dnmt3a*-mutant HSCs have an increased competitive advantage in middle-aged recipients^14^, we employed a defined genetic model of IGF1 deficiency that mimics aspects of the middle-aged BM microenvironment^17,18^. We first evaluated whether *Dnmt3a*-mutant HSCs also have a competitive advantage in an *Igf1*-deficient environmental context using a competitive bone marrow transplant. Donor CD45.2^+^ BM cells from a mouse model of human *DNMT3A*-mutant hematopoiesis (*Dnmt3a*^R878H/+^ Mx-Cre)^19^ or Mx-Cre control BM cells were mixed with wild-type CD45.1^+^ BM cells at a 1:1 ratio. This mixture was transplanted into *Igf1* conditional knockout recipient mice (*Igf1*^fl/fl^ Cre-ER^T2^) or control recipient mice (*Igf1*^+/+^ Cre-ER^T2^) (Fig. 1A). *Dnmt3a*^R878H/+^ hematopoietic cells had an increased competitive advantage over wild-type cells in *Igf1*-deficient recipient mice in the peripheral blood at 28 weeks post-transplant (Fig. 1**B**). *Igf1*-deficient recipients also had increased frequency and total number of *Dnmt3a*^R878H/+^ HSCs in the bone marrow (Fig. 1C, Supplementary Fig. 1a). Unlike Mx-Cre control cells, *Dnmt3a*^R878H/+^ hematopoietic cells in *Igf1*-deficient recipients maintained a balanced mature cell lineage composition (Supplementary Fig. 1B–D), suggesting the competitive advantage of *Dnmt3a*^R878H/+^ hematopoiesis in *Igf1*-deficient recipients is established at the level of multipotent HSCs rather than expansion of lineage-biased progenitor cells.

Based on our previous work^18^, we hypothesized that *Dnmt3a*^R878H/+^ HSCs may have a competitive advantage in aging and *Igf1*-deficient environments due to differential regulation of metabolism. Using Seahorse extracellular metabolic flux assays (Fig. 1D), we observed that unstimulated *Dnmt3a*^R878H/+^ HSPCs had higher maximal respiration and spare respiratory capacity compared to unstimulated control HSPCs (Fig. 1E, F). A similar signal was observed in control HSPCs in response to IGF1 whereas the elevated respiration in *Dnmt3a*^R878H/+^ HSPCs was not further increased in response to IGF1. The phenotype of increased maximal respiration and spare respiratory capacity in unstimulated *Dnmt3a*^R878H/+^ HSPCs compared to control HSPCs was confirmed in independent experiments (Fig. 1G, H), and no change in glycolytic capacity was observed between *Dnmt3a*^R878H/+^ and control HSPCs (Fig. 1I). To examine whether this phenotype was exclusive to *Dnmt3a*-mutant hematopoiesis or conserved among CH-associated mutations, we evaluated *Tet2*^−/−^ and control HSPCs. We observed that *Tet2*^−/−^ HSPCs also had increased basal respiration and maximal respiration (Supplementary Fig. 1e, f), consistent with the *Dnmt3a*^R878H/+^ phenotype. As these phenotypes were evaluated after ex vivo culture, we also tested *Dnmt3a*^R878H/+^ HSPCs isolated directly from mice (Fig. 1J). De novo isolated *Dnmt3a*^R878H/+^ HSPCs had increased maximal cellular respiration (Fig. 1K) and increased spare respiratory capacity (Fig. 1L) compared to de novo isolated control HSPCs.

### *Dnmt3a*-mutant HSPCs exhibit elevated mitochondrial membrane potential associated with DNA hypomethylation and increased expression of electron transport chain complex and supercomplex machinery

**Fig. 2:**
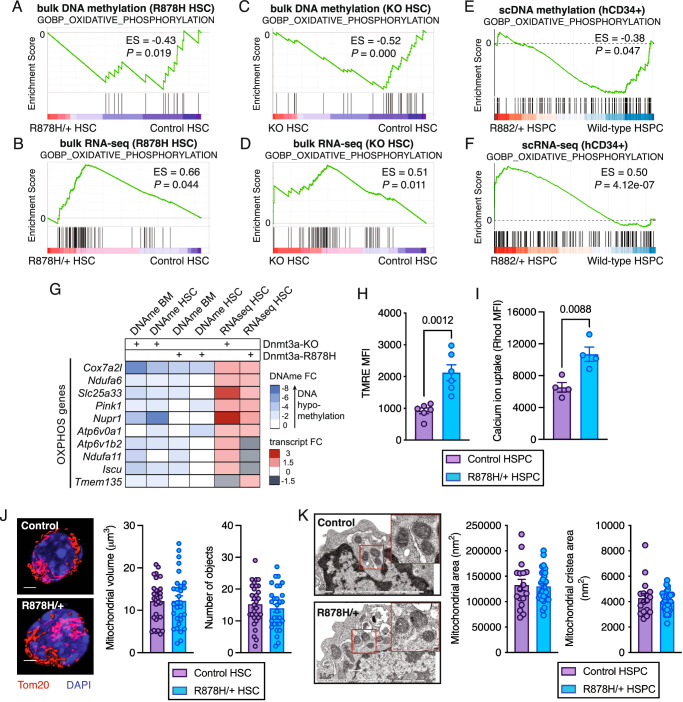
DNA hypomethylation and increased expression of electron transport chain machinery and elevated mitochondrial membrane potential in Dnmt3a-mutant HSPCs. **A**–**F** Enrichment analysis of an oxidative phosphorylation gene signature in DNA methylation data (**A**, **C**, **E**) and RNA-seq data (**B**, **D**, **F**) comparing *Dnmt3a*^R878H/+^ vs. control HSC (**A**, **B**), *Dnmt3a* KO versus control HSC (**C**, **D**), *DNMT3A*^R882^ CD34^+^ cells versus *DNMT3A*^+/+^ CD34^+^ cells (**E**, **F**). Includes data from Jeong et al^20^ and Nam et al.^22^. **G** DNA methylation and expression heatmap of oxidative phosphorylation genes in *Dnmt3a*-KO and *Dnmt3a*^R878H/+^ BM and HSCs. Includes data from Li et al^21^ and Jeong et al.^20^. **H** TMRE mean fluorescence intensity in control and *Dnmt3a*^R878H/+^ HSCs. Bars represent mean ± SEM with points from biological replicate mice (*n* = 6). Statistical analysis used unpaired, two-tailed *t* test. **I** Calcium ion uptake in control and *Dnmt3a*^R878H/+^ HSCs. Bars represent mean ± SEM with points from biological replicate mice (*n* = 4). Statistical analysis used unpaired, two-tailed *t* test. **J** Representative images (left) of control and *Dnmt3a*^R878H/+^ HSCs stained with TOM20 (red) and DAPI (blue). Scale bar denotes 2 μm. Mitochondrial volume (center) and mitochondrial number (right) in control and *Dnmt3a*^R878H/+^ HSCs. Bars represent mean ± SEM with data points from biological replicate HSCs (*n* = 30) from three mice per genotype. Statistical analysis used unpaired, two-tailed *t* test. **K** Representative transmission electron microscopy images (left) of control and *Dnmt3a*^R878H/+^ HSPCs. Scale bar denotes 0.5 μm. Red squares denote zoomed region. Mitochondrial area (center) and mitochondrial cristae area (right) in control and *Dnmt3a*^R878H/+^ HSPCs. Bars represent mean ± SEM with points are from biological replicate HSPCs (*n* = 23 control HSPC, *n* = 32 R878H/ + HSPC) from three mice per genotype. Statistical analysis used unpaired, two-tailed *t* test. Source data are provided as a Source Data file.

To evaluate if enhanced mitochondrial cellular respiration in *Dnmt3a*^R878H/+^ HSPCs was associated with loss of DNA methyltransferase function of the Dnmt3a protein and DNA hypomethylation, we performed whole genome bisulfite sequencing of *Dnmt3a*^R878H/+^ and control HSCs. Genes with DNA hypomethylation in *Dnmt3a*^R878H/+^ HSCs were enriched for an oxidative phosphorylation gene signature (Fig. 2A). We also examined published transcriptome and DNA methylation datasets from mouse *Dnmt3a*^R878H/+^ HSCs and *Dnmt3a* knockout HSCs^20,21^. In all cases, genes with DNA hypomethylation and increased transcript expression were enriched for the same oxidative phosphorylation gene signature (Fig. 2B–D). Analysis of single cell DNA methylation and transcriptome data from human *DNMT3A*^R882^-mutant CH^22^ revealed DNA hypomethylation and increased expression of oxidative phosphorylation genes in *DNMT3A*^R882^ CD34^+^ cells compared to *DNMT3A*^+/+^ CD34^+^ cells in the same individuals (Fig. 2E, F). These data support that the enhanced molecular signatures of oxidative phosphorylation in *Dnmt3a*-mutant and *Dnmt3a*-knockout HSPCs are conserved between mouse and human and are associated with DNA hypomethylation.

To explore the mechanisms by which DNA hypomethylation relates to increased oxidative phosphorylation, we examined four independent DNA methylation datasets and two transcriptome datasets comparing *Dnmt3a*^R878H/+^ or *Dnmt3a*-knockout BM cells and HSCs to their control counterparts^20,21^. Genes with both DNA hypomethylation and increased expression included mediators of electron transport chain supercomplexes (*Cox7a2l*, *Ndufa6, Pink1, Ndufa11*), ETC complex V activity (*Atp6v0a1, Atp6v1b2*) and mitochondrial DNA and RNA synthesis (*Slc25a33*) (Fig. 2G). *Cox7a2l* was the only gene hypomethylated and increased in expression across all datasets. By mediating the assembly of respiratory chain supercomplexes, Cox7a2l is essential for mitochondrial efficiency and mitochondrial membrane potential (Δψm)^23–26^. We observed that *Dnmt3a*^R878H/+^ HSCs have increased Δψm compared to control HSCs by functional analysis using tetramethyl rhodamine ethyl ester (TMRE) (Fig. 2H). Consistent with increased Δψm, *Dnmt3a*^R878H/+^ HSCs also exhibited enhanced uptake of positively charged calcium ions (Fig. 2I). *Dnmt3a*^R878H/+^ HSCs did not have alterations in volume or number of mitochondria assessed by immunofluorescence staining (Fig. 2J), nor gross changes mitochondrial morphology, area, or cristae area assessed by electron microscopy (Fig. 2K). *Dnmt3a*^R878H/+^ and control HSPCs also had similar mitochondrial DNA content (Supplementary Fig. 2). Together, these data suggest that enhanced mitochondrial cellular respiration observed in *Dnmt3a*^R878H/+^ HSPCs is a consequence of elevated Δψm rather than altered mitochondrial morphology or number.

### Elevated mitochondrial membrane potential sensitizes *Dnmt3a*-mutant HSPCs to inhibition of the electron transport chain

**Fig. 3:**
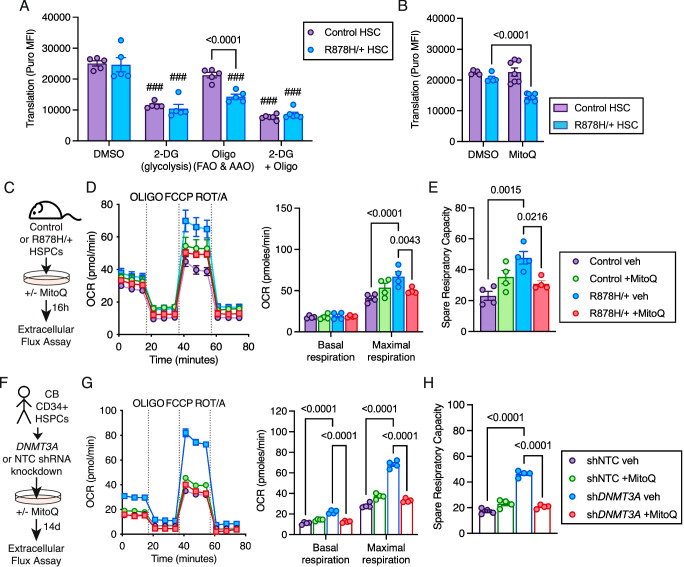
Mouse and human *Dnmt3a*-mutant HSPCs are sensitive to electron transport chain inhibitors. **A** Quantification of puromycin incorporation in control and *Dnmt3a*^R878H/+^ HSCs treated with vehicle control (DMSO), 2-deoxygluocse (2-DG), oligomycin (Oligo) or 2-DG + Oligo using SCENITH assay. Bars represent mean ± SEM with points from biological replicate mice (*n* = 5 DMSO, *n* = 5 2-DG, *n* = 5 Oligo, *n* = 6 2-DG+Oligo). Statistical analyses used two-way ANOVA with Tukey’s multiple comparisons test. **B** Quantification of metabolic function in control and *Dnmt3a*^R878H/+^ HSCs after treatment with MitoQ using SCENITH assay. Bars represent mean ± SEM with points from biological replicate mice (*n* = 4 DMSO, *n* = 7 MitoQ). Statistical analyses used two-way ANOVA with Dunnett’s multiple comparisons test. **C** Experimental design. **D** OCR profile plot (left) used to quantify basal and maximal respiration (right) and (**E**), spare respiratory capacity. **D**, **E** Symbols represent mean ± SEM, bars represent mean ± SEM with points from biological replicate mice (*n* = 4). Statistical analyses used two-way ANOVA with Tukey’s multiple comparisons test (**D**) and one-way ANOVA with Tukey’s multiple comparisons test (**E**). **F** Experimental design. **G** OCR profile plot (left) used to quantify basal and maximal respiration (right) and (**H**), spare respiratory capacity. **F**–**H** Symbols represent mean ± SEM, bars represent mean ± SEM with points from biological replicates (*n* = 4). Statistical analyses used two-way ANOVA with Tukey’s multiple comparisons test (**G**) and one-way ANOVA with Fisher’s LSD (**H**). Source data are provided as a Source Data file.

To assess the dependency of *Dnmt3a*^R878H/+^ HSCs on cellular respiration, we utilized the SCENITH assay (single cell energetic metabolism by profiling translation inhibition)^27^ which quantifies changes in protein translation as a measure of metabolic activity in response to metabolic inhibitors. We found that *Dnmt3a*^R878H/+^ HSCs have greater dependence on oligomycin-targetable oxidative phosphorylation compared to control HSCs (Fig. 3A) and have reduced glycolytic capacity when mitochondrial oxidative phosphorylation is inhibited (Supplementary Fig. 3a–d). Enhanced electron transport chain function and supercomplex formation are targetable by lipophilic triphenylphosphonium (TPP^+^) molecules, including the small molecule mitoquinol (MitoQ)^28^. Similar to the effects of oligomycin, we found that MitoQ decreased metabolic activity in *Dnmt3a*^R878H/+^ HSCs but not in control HSCs (Fig. 3B). As MitoQ has been shown to have beneficial effects on enhancing wild-type HSC function in the context of aging^29^, we pursued this molecule for further study. Using Seahorse extracellular metabolic flux assays (Fig. 3C), MitoQ treatment decreased the maximal respiration (Fig. 3D) and spare respiratory capacity (Fig. 3E) of *Dnmt3a*^R878H/+^ HSPCs and did not impact control HSPCs. Evaluating this phenotype in human cells, primary cord blood (CB) CD34^+^ HSPCs with shRNA knockdown of *DNMT3A* (Fig. 3F) had increased basal respiration (Fig. 3G), maximal respiration, and spare respiratory capacity (Fig. 3H). MitoQ treatment decreased these parameters in *DNMT3A* knockdown CD34^+^ HSPCs to levels consistent with control HSPCs. Thus, elevated mitochondrial cellular respiration in *Dnmt3a*-mutant HSPCs is conserved between mouse and human and represents a selective molecular dependency that can be targeted using electron transport chain inhibitors including MitoQ.

### Elevated Δψm in *Dnmt3a*-mutant HSPCs is linked to greater uptake and accumulation of long-chain alkyl-TPP molecules

**Fig. 4:**
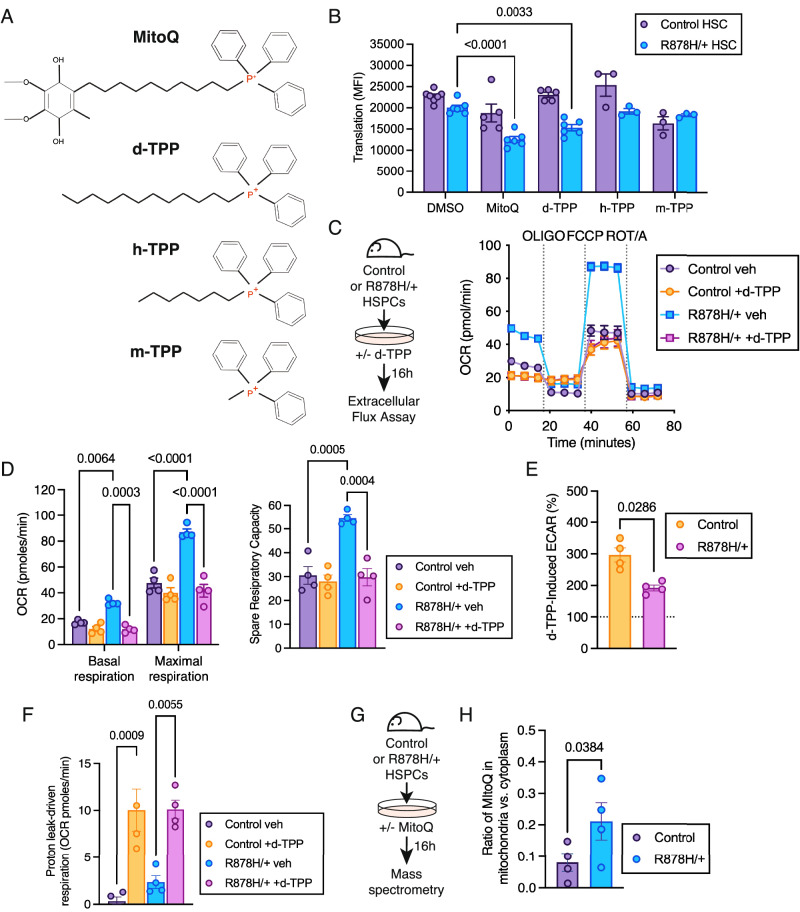
*Dnmt3a*^R878H/+^ HSPCs have greater uptake and accumulation of long-chain alkyl-TPP molecules. **A** Chemical structures of TPP^+^ derivatives MitoQ, d-TPP, h-TPP and m-TPP. Created in BioRender: https://BioRender.com/j69r060. **B** Quantification of puromycin incorporation in control and *Dnmt3a*^R878H/+^ HSCs treated with vehicle control (DMSO), MitoQ, d-TPP, h-TPP, or m-TPP. Bars represent mean ± SEM, points from biological replicate mice (*n* = 7 Control DMSO, *n* = 6 R878H/ + DMSO, *n* = 5 Control MitoQ, *n* = 6 R878H/+ MitoQ, *n* = 5 Control d-TPP, *n* = 6 R878H/+ d-TPP, *n* = 3 Control h-TPP, *n* = 3 R878H/+ h-TPP, *n* = 3 Control m-TPP, *n* = 3 R878H/+ m-TPP). Statistical analysis used two-way ANOVA with Dunnett’s multiple comparisons test. **C** Experimental design (left) and OCR plot (right) used to quantify (**D**), basal and maximal respiration (left) and spare respiratory capacity (right) in d-TPP treated control and *Dnmt3a*^R878H/+^ HSPCs. **E** Quantification of d-TPP induced ECAR in d-TPP treated control and *Dnmt3a*^R878H/+^ HSPCs. **F** Proton-leak driven respiration in d-TPP treated control and *Dnmt3a*^R878H/+^ HSPCs. **D**–**F** Bars represent mean ± SEM, points are from biological replicate mice (*n* = 4). Statistical analyses used two-way ANOVA with Sidak’s multiple comparisons test (**D**, left), one-way ANOVA with Tukey’s multiple comparisons test (**D**, right, **F**), and unpaired, two-tailed *t* test (**E**). **G** Experimental design. **H** Ratio of MitoQ in the mitochondria vs. cytoplasm of control and *Dnmt3a*^R878H/+^ HSPCs. Bars represent mean ± SEM, points from biological replicate mice (*n* = 4). Statistical analysis used ratio paired, two-tailed *t* test. Source data are provided as a Source Data file.

MitoQ consists of a lipophilic cation (triphenylphosphonium [TPP]) coupled to an antioxidant component (quinone) by a 10-carbon alkyl chain (Fig. 4A). Based on this structure, a potential mechanism by which MitoQ selectively targets *Dnmt3a*^R878H/+^ HSPCs is by accumulation in the mitochondrial matrix due to increased Δψm in these cells. As mitochondrial accumulation decreases with decreased alkyl chain length^30^, we tested how TPP conjugates with varying carbon chain lengths impact protein translation as a proxy for metabolic activity. We evaluated carbon alkyl chain lengths of 12 (dodecyl-TPP [d-TPP]), 7 (heptyl-TPP [h-TPP]) and 1 (methyl-TPP [m-TPP]) (Fig. 4A) compared to the positive control MitoQ. We observed that MitoQ and d-TPP reduced protein translation/metabolic activity in *Dnmt3a*^R878H/+^ HSCs to a similar level while the shorter TPP conjugates h-TPP and m-TPP did not have a significant effect (Fig. 4B). Using Seahorse extracellular metabolic flux assays (Fig. 4C), d-TPP treatment of *Dnmt3a*^R878H/+^ HSPCs decreased their basal and maximal cellular respiration and spare respiratory capacity (Fig. 4D) to levels comparable to untreated control HSPCs. As longer-chain alkyl-TPP molecules have been reported to have additional effects of enhancing glycolysis and increasing proton leak^30^, we assessed these phenotypes following d-TPP treatment. Consistent with the reduced glycolytic capacity of *Dnmt3a*^R878H/+^ HSPCs when oxidative phosphorylation is inhibited (Supplementary Fig. 3b), *Dnmt3a*^R878H/+^ HSPCs treated with d-TPP have a reduced ability to undergo glycolysis (Fig. 4E). In addition, d-TPP treatment caused proton leak-driven cellular respiration to a similar degree in control and *Dnmt3a*^R878H/+^ HSPCs (Fig. 4F), supporting that this does not underlie differential sensitivity of *Dnmt3a*^R878H/+^ HSPCs to long-chain alkyl-TPP molecules. To formally evaluate accumulation in the mitochondria, we performed mass spectrometry-based quantitation of MitoQ in mitochondrial lysates and cytoplasmic lysates (Fig. 4G). This analysis revealed greater accumulation of MitoQ in the mitochondria of *Dnmt3a*^R878H/+^ HSPCs compared to control HSPCs (Fig. 4H). Thus, elevated Δψm of *Dnmt3a*-mutant HSPCs can be harnessed using long-chain alkyl-TPP molecules to specifically target enhanced mitochondrial cellular respiration of *Dnmt3a*-mutant HSPCs.

### MitoQ induces the mitochondrial apoptotic pathway selectively in *Dnmt3a*-mutant HSPCs

**Fig. 5:**
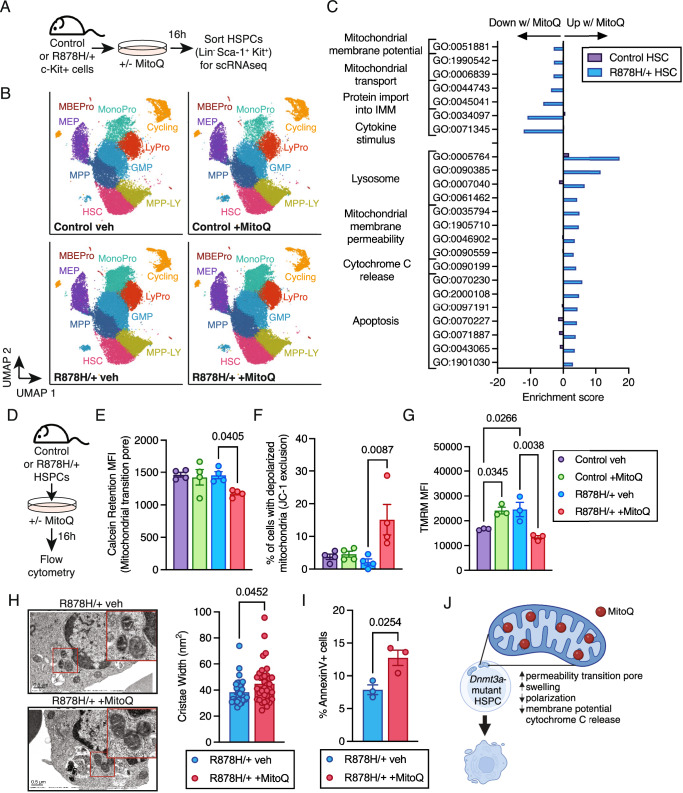
MitoQ induces activation of the mitochondrial apoptotic pathway selectively in *Dnmt3a*^R878H/+^ HSPCs. **A** Experimental design for single-cell RNA-seq from biological replicate mice (*n* = 4). **B** UMAP plots of scRNA-seq showing 9 cell clusters from a total of 134,844 cells. **C** Negative and positive enrichment of gene ontology terms in control and *Dnmt3a*^R878H/+^ HSCs treated with MitoQ vs. vehicle. **D** Experimental design. **E** Quantification of loss of calcein retention in control and *Dnmt3a*^R878H/+^ HSCs treated with MitoQ vs. vehicle. **F** Quantification of JC-1 exclusion in control and *Dnmt3a*^R878H/+^ HSC treated with MitoQ vs. vehicle. **E**, **F** Bars represent mean ± SEM, points from biological replicate mice (*n* = 4). Statistical analyses used one-way ANOVA with Sidak’s multiple comparisons test. **G** Quantification of mitochondrial membrane potential in control and *Dnmt3a*^R878H/+^ HSC treated with MitoQ vs. vehicle. Bars represent mean ± SEM, points from biological replicate mice (*n* = 3). Statistical analysis used one-way ANOVA with Sidak’s multiple comparisons test. **H** Representative images (left) of *Dnmt3a*^R878H/+^ HSPCs treated with MitoQ or vehicle using TEM. Scale bar denotes 0.5 μm. Red squares denote zoomed area. Quantification of mitochondrial cristae width (right) in *Dnmt3a*^R878H/+^ HSPCs treated with MitoQ or vehicle. Bars represent mean ± SEM, points from biological replicate HSPCs (*n* = 32 R878H/+ veh, *n* = 35 R878H/+ +MitoQ) from three mice per genotype. Statistical analysis used unpaired, two-tailed *t* test. **I** Frequency of AnnexinV+ *Dnmt3a*^R878H/+^ HSPCs after treatment with MitoQ or vehicle. Bars represent mean ± SEM, points from biological replicate mice (*n* = 3). Statistical analysis used unpaired, two-tailed *t* test. **J** Model of MitoQ uptake in *Dnmt3a*^R878H/+^ HSPCs leading to increased permeability transition pore, increased swelling, depolarization, decreased MMP, cytochrome C release and apoptosis. Created in Biorender: https://BioRender.com/m381057. Source data are provided as a Source Data file.

We next sought to understand the molecular and cellular consequences of mitochondrial accumulation of long-chain alkyl-TPP molecules in *Dnmt3a*^R878H/+^ HSPCs. We profiled the transcriptional effects of MitoQ on *Dnmt3a*^R878H/+^ HSPCs using single cell RNA-seq (Fig. 5A). Clustering and cell type annotation identified 9 HSPC subsets (Fig. 5B) and differential expression analysis was performed on each cluster (Supplementary Fig. 4a). We focused first on the transcriptional changes induced by MitoQ in *Dnmt3a*^R878H/+^ HSCs that were not induced in control HSCs. MitoQ treatment decreased expression of molecular signatures of Δψm, mitochondrial transport and import into the inner mitochondrial membrane in *Dnmt3a*^R878H/+^ HSCs (Fig. 5C), consistent with the reduced cellular respiration phenotypes we observed. In addition, MitoQ-treated *Dnmt3a*^R878H/+^ HSCs had increased expression of molecular signatures of the lysosome, including increased expression of Cathepsin B (*Ctsb)* which is involved in apoptotic cell death (Supplementary Fig. 4b), mitochondrial membrane permeability, cytochrome C release and apoptosis (Supplementary Fig. 4c). These signatures were also significantly increased in other *Dnmt3a*^R878H/+^ HSPC populations such as MPP, GMP, MonoPro and LyPro (Supplementary Fig. 4d), supporting that MitoQ impacts multiple populations within the HSPC compartment. In contrast, transcriptional changes induced by MitoQ in control HSCs reflected reduced reactive oxygen species and increased mitochondrial function (Supplementary Fig. 4e, f), consistent with MitoQ being an antioxidant that has a beneficial effect on metabolism and function of aged wild-type HSCs^29,31^. These data support fundamentally distinct molecular actions of MitoQ in *Dnmt3a*^R878H/+^ HSPCs compared to control HSPCs.

We then tested candidate mechanisms nominated by our molecular analysis at the cellular level (Fig. 5D). MitoQ treatment of *Dnmt3a*^R878H/+^ HSPCs resulted in opening of the mitochondrial transition pore, an early step in the mitochondrial apoptotic pathway, indicated by reduced retention of the mitochondrially-localized molecule calcein (Fig. 5E). Further, MitoQ treatment of *Dnmt3a*^R878H/+^ HSPCs caused mitochondrial depolarization (Fig. 5F), reduced Δψm (Fig. 5G), mitochondrial swelling (Fig. 5H), and increased frequency of apoptotic cells assessed by Annexin V (Fig. 5I). MitoQ reduced the proportion of control HSPCs in cell cycle but did not impact cycling of *Dnmt3a*^R878H/+^ HSPCs (Supplementary Fig. 4g). Thus, molecular and cellular phenotypes support that MitoQ reduces cellular respiration and can induce the mitochondrial apoptotic pathway selectively in *Dnmt3a*-mutant HSPCs (Fig. 5J).

### Long-chain alkyl-TPP molecules ablate the competitive advantage of *Dnmt3a*-mutant HSPCs ex vivo and in vivo

**Fig. 6:**
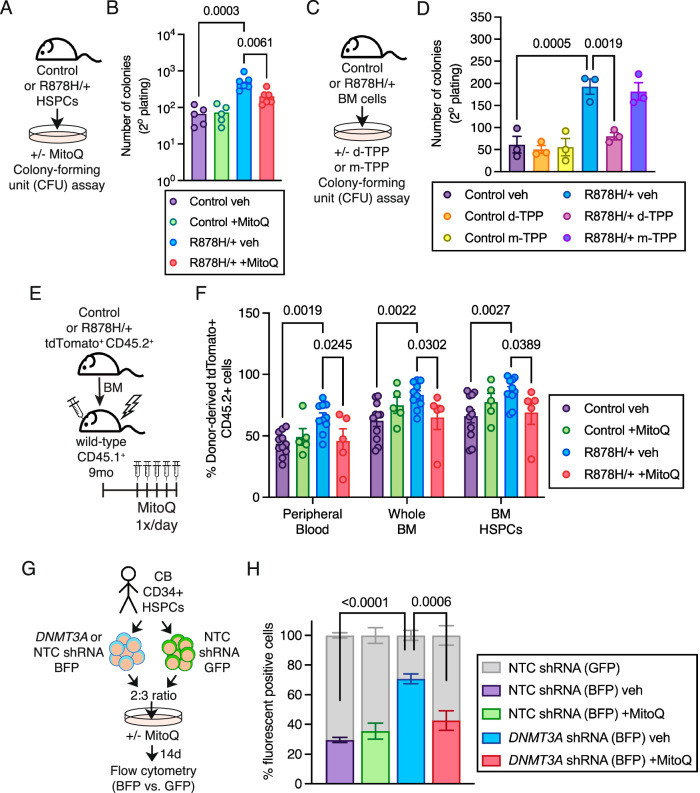
Long-chain alkyl-TPP molecules ablate the competitive advantage of mouse and human *Dnmt3a*-mutant HSPCs and their progeny. **A** Experimental design. **B** Number of myeloid CFU in secondary plating of control and *Dnmt3a*^R878H/+^ BM cells. Bars represent mean ± SEM, points from biological replicate mice (*n* = 5 control veh, *n* = 5 control +MitoQ, *n* = 6 R878H/+ veh, *n* = 6 R878H/+ +MitoQ). Statistical analysis used one-way ANOVA with Sidak’s multiple comparisons test. **C** Experimental design. **D** Number of myeloid CFU in secondary plating of control and *Dnmt3a*^R878H/+^ BM cells. Bars represent mean ± SEM, points from biological replicate mice (*n* = 3). Statistical analysis used one-way ANOVA with Sidak’s multiple comparisons test. **E** Experimental design. **F** Frequency of donor-derived tdTomato^+^ CD45.2^+^ cells in PB, BM and HSPCs of control and *Dnmt3a*^R878H/+^ recipient mice after treatment with MitoQ. Bars represent mean ± SEM, points from biological replicate mice (*n* = 11 control veh, *n* = 5 control +MitoQ, *n* = 10 R878H/+ veh, *n* = 5 R878H/+ +MitoQ). The transplant was performed 3 independent times. Statistical analysis used two-way ANOVA with Fisher’s LSD test. **G** Experimental design. **H** Frequency of *DNMT3A-*knockdown human CD34+ cells after 14 days of MitoQ treatment. Bars represent mean ± SEM, points are from one human sample with technical replicates (*n* = 6). Statistical analysis used two-way ANOVA with Tukey’s multiple comparisons test. Source data are provided as a Source Data file.

We evaluated the functional consequences of long-chain alkyl-TPP molecules on the competitive advantage of *Dnmt3a*^R878H/+^ HSPCs and their progeny. First, we used ex vivo serial colony-forming unit (CFU) assays to assess the growth advantage of HSPCs (Fig. 6A). MitoQ treatment decreased the secondary CFU replating of *Dnmt3a*^R878H/+^ HSPCs but had no effect on control HSPCs (Fig. 6B). Treatment with d-TPP (Fig. 6C) had a similar effect of decreased secondary CFU replating of *Dnmt3a*^R878H/+^ HSPCs while the short form m-TPP had no effect (Fig. 6D).

We then assessed the in vivo effects on MitoQ based on our reported physiologically relevant observation that *Dnmt3a*-mutant HSCs have an enhanced competitive advantage in middle-aged recipient mice^14^. We transplanted control or *Dnmt3a*^R878H/+^ BM cells into 9-month-old recipient mice, allowed these recipients to age to 12 months old, and administered MitoQ in vivo for five consecutive days^29,32^ (Fig. 6E). As expected, *Dnmt3a*^R878H/+^ cells had greater contribution to hematopoiesis compared to control cells in middle-aged recipients assessed at the peripheral blood, bone marrow and HSPC levels (Fig. 6F). Treatment of middle-aged mice with MitoQ decreased the contribution of *Dnmt3a*^R878H/+^ cells to hematopoiesis at all cellular levels, mirroring the proportions of control cells. To evaluate this phenotype in primary human cells, ex vivo cultures of cord blood (CB) CD34^+^ HSPCs with *DNMT3A* knockdown were competed against control CD34^+^ HSPCs at a 2:3 ratio (Fig. 6G). In vehicle-treated control cultures, we observed a clear competitive advantage of *DNMT3A* knockdown human HSPCs (Fig. 6H). The addition of MitoQ significantly reduced the competitive growth advantage of *DNMT3A* knockdown HSPCs. Thus, long-chain alkyl-TPP molecules such as MitoQ can be used to impair the competitive growth advantage of mouse and human *DNMT3A*-mutant HSPCs, reducing the burden of clonal hematopoiesis.

## Discussion

Here we have shown that *Dnmt3a*-mutant HSPCs achieve a selective advantage over wild-type HSPCs through elevated mitochondrial membrane potential and cellular respiration. Associated with DNA hypomethylation and increased expression of components of the electron transport chain and respiratory supercomplex formation, *Dnmt3a*-mutant HSPCs have an elevated Δψm and greater dependency on mitochondrial respiration which sensitizes them to inhibition of the electron transport chain. Greater uptake and accumulation of long-chain alkyl-TPP molecules selectively reduce mitochondrial respiration in *Dnmt3a*-mutant HSPCs, induce the mitochondrial apoptotic pathway, and ablate the competitive advantage of *Dnmt3a*-mutant HSPCs and their progeny. Importantly, key observations were reproduced in human *DNMT3A*-knockdown HSPCs, supporting species conservation of this mechanism and translational relevance of our findings.

In wild-type HSCs, Δψm is positively associated with self-renewal and regenerative capacity, and Δψm is reduced with HSC dysfunction as a consequence of aging and post-chemotherapy^18,33^. Our data suggests that *Dnmt3a*-mutant HSPCs have increased Δψm as a consequence of DNA hypomethylation and increased gene expression of components of the electron transport chain and respiratory supercomplex formation. Interestingly, we and others have observed enhanced oxidative phosphorylation and cellular respiration as a consequence of other recurrent CH mutations including *Tet2, Asxl1*^34^, and *Idh1*^35^ although the mechanisms causing these phenotypes remain to be discerned. Taken together, Δψm and enhanced mitochondrial respiration may be a common mechanism by which stem cells have a competitive survival advantage in the context of aging, inflammation or stress environments.

Targeting strategies based on mitochondrial accumulation of lipophilic, positively charged long-chain alkyl-TPP molecules (MitoQ, d-TPP) were able to successfully reduce Δψm and mitochondrial respiration in *Dnmt3a*-mutant HSPCs. In addition to disrupting respiratory supercomplex formation^28^, these molecules destabilize the mitochondrial membrane and inhibit ETC complexes I, II, III and IV in a dose-dependent manner^36^. To what extent mitochondrially targeted compounds reduce mitochondrial respiration in *Dnmt3a*-mutant HSPCs by mitochondrial matrix destabilization versus direct inhibition of ETC activity or supercomplex activity will require additional investigation currently limited by the lack of techniques adapted for low input cell number. In addition to reduced mitochondrial respiration, our observation that MitoQ induces the mitochondrial apoptotic pathway in a subset of *Dnmt3a*-mutant HSPCs also contributes to the efficacy of this molecule in reducing *Dnmt3a*-mutant hematopoiesis.

From a therapeutic standpoint, given that CH is not an overt disease state, the only reasonable interventions should be efficacious at a dose and duration with no toxicity and low to no side effects. TPP-based compounds can be given to rodents at high doses without significant damage to organs including the heart, liver and kidneys^37^. MitoQ and d-TPP have been administered continuously to mice in drinking water for 28 weeks with no adverse events including no effect on whole-body metabolism, motor function, and food or liquid consumption^38^. In humans, MitoQ has been administered orally to patients in Phase II studies from 28 days to 6 weeks duration with no significant adverse side effects^39–42^. Further studies are needed to determine the dose at which systemic administration of MitoQ or d-TPP would be effective in reducing the selective advantage of *Dnmt3a*-mutant HSPCs, the durability of the treatment effect, and side effect(s) observed with that treatment regimen. Apart from TPP-based compounds, FDA-approved molecules that reduce electron transport chain function and mitochondrial respiration such as metformin, tamoxifen, and diclofenac^43^ may also have utility in selectively targeting CH-mutant HSPCs. An intriguing aspect of targeting CH-mutant cells with molecules like MitoQ is that this same molecule has been shown through its antioxidant function to enhance wild-type aged HSC function and boost adaptive immune cell production^44^. Thus, it is conceivable that a single mitochondrially-targeted molecule could be employed to increase the contribution of wild-type HSPCs to hematopoiesis and lymphopoiesis in aged individuals while decreasing clonal hematopoiesis, therein theoretically reducing risk of aging- and CH-associated disease.

## Methods

### Experimental animals

B6.SJL-*Ptprc*^*a*^*Pepc*^*b*^/BoyJ mice^45^ (referred to as CD45.1) were obtained from, and aged within, The Jackson Laboratory. B6.129(FVB)-*Igf1*^*tm1Dlr*^/J mice^46^ (referred to as Igf1^fl/fl^) were crossed to B6.129-*Gt(ROSA)26Sor*^*tm1(cre/ERT2)Tyj*^/J mice^47^ (referred to as Cre-ER^T2^). *Dnmt3a*^fl-R878H/+^ mice^19^ were crossed to B6.Cg-Tg (Mx1-cre)1Cgn/J mice^48^ (referred to as Mx-Cre) or C57BL/6N-Fgd*5*t^m3(cre/ERT2)Djr^/J mice^49^ (referred to as Fgd5-CreER^T2^) crossed to B6.Cg-Gt-ROSA)26Sor^tm14(CAG-tdTomato)Hze^/J mice^50^ (referred to as LSL-tdTomato)^50^. Tet2^fl/fl^ mice^51^ were crossed to Vav-Cre mice^52^. All experiments used Cre-only mice as a control group, adult (2–6 months of age) female and male mice were used throughout these studies. Mice are housed in cages with same sex, 12 hr/12 hr light and dark cycle with controlled temperature and humidity. All strains were obtained from The Jackson Laboratory repository. To induce Mx1-Cre, mice were intraperitoneally (IP) injected once every other day for five total injections with 15 mg/kg high molecular weight polyinosinic-polycytidylic acid (InvivoGen). To induce Cre-ER^T2^ or Fgd5-CreER^T2^, mice received 125 mg/kg tamoxifen for three consecutive days by oral gavage. All mouse work was approved by The Animal Care and Use Committee at The Jackson Laboratory.

### In vivo transplantation

For transplantation experiments into Igf1^fl/fl^ Cre-ER^T2^ recipient mice, 1 x 10^6^ mononuclear cells (MNCs) from donor CD45.1 mice and 1 x 10^6^ MNCs from donor *Dnmt3a*^+/+^ Mx-Cre or *Dnmt3a*^R878H/+^ Mx-Cre mice were transplanted into lethally irradiated *Igf1*^+/+^ Cre-ER^T2^ mice or *Igf1*^fl/fl^ Cre-ER^T2^ recipient mice. Injection of tamoxifen (TAM) to induce *Igf1* knockout was performed 4 weeks after transplant to avoid confounding responses to irradiation^53^. For transplantation experiments into middle-aged CD45.1 recipient mice, 3 × 10^6^ MNCs from *Dnmt3a*^+/+^ Fgd5-CreER^T2^ LSL-tdTomato or *Dnmt3a*^R878H/+^ Fgd5-CreER^T2^ LSL-tdTomato donor mice were transplanted into 9-month-old busulfan-conditioned (20 mg/kg/per day for consecutive 3 days, Sigma-Aldrich B2635) CD45.1 recipient mice.

### Peripheral blood analysis

Blood was collected from mice via the retro-orbital sinus and red blood cells were lysed using 1X RBC lysis buffer. Remaining MNCs were stained with fluorochrome-conjugated antibodies recognizing CD45.1 (clone A20, Biolegend, 1:200), CD45.2 (clone 104, Biolegend, 1:50), B220 (clone RA3-6B2, BD Biosciences, 1:200), CD3e (clone 145-2C11, BD Biosciences, 1:200), CD11b (clone M1/70, Biolegend, 1:200), Ter-119 (clone TER-119, Biolegend, 1:50), Gr-1 (clone RB6-8C5, Biolgend, 1:200), Ly6g (clone 1A8, Biolegend, 1:200), Ly6c (clone HK1.4, Biolegend, 1:200) and F4/80 (clone BM8, Invitrogen, 1:200). DAPI was used as a viability stain. Data was captured using a LSRII (BD) and analyzed using FlowJo V10.

### Isolation of hematopoietic stem and progenitor cells

BM cells were isolated from pooled and crushed femurs, tibiae, and iliac crests of individual mice. Following red blood cell lysis using 1X RBC lysis buffer (eBioscience), BM MNCs were stained with a combination of fluorochrome-conjugated antibodies recognizing c-Kit (clone 2B8, Biolegend, 1:50), Sca-1 (clone D7, Biolegend, 1:200), CD150 (clone TC15-12F12.2, Biolegend, 1:200), CD48 (clone HM48-1, Biolegend, 1:200), CD34 (clone RAM34, BD Biosciences, 1:100), Flt3 (clone A2F10, Biolegend, 1:50), and a mature lineage (Lin) marker mix including B220 (clone RA3-6B2, Biolegend, 1:200), CD11b (clone M1/70, Biolegend, 1:200), CD4 (clone RM4-5, Biolegend, 1:200), CD5 (clone 53-7.3, Biolegend, 1:200), CD8a (clone 53-6.7, Biolegend, 1:200), Ter-119 (clone TER-119, Biolegend, 1:200) and Gr-1 (clone RB6-8C5, Biolgend, 1:200). DAPI was used as a viability stain. HSC isolation and/or phenotyping was based on the surface markers Lin- Sca-1+ c-Kit+ Flt3- CD150 + CD48- using a FACSAria II, FACSymphony S6 or FACSymphony A5 (BD). HSPCs were isolated by incubating BM cells with c-Kit microbeads (Miltenyi Biotec, 130-091-224), washing with MACS Buffer and enriched using an AutoMACS Pro cell separator (Miltenyi Biotec). Additionally, HSPCs were isolated by sortwelcomefor Lin- Sca-1+ c-Kit+ population. For experiments using Ki67 BM cells were fixed and permeabilized using the eBioscience Foxp3/Transcription Factor Staining Buffer Set (ThermoFisher) per the manufacturer’s instructions. Stained cells were sorted using a FACSAria II or a FACSymphony S6 Sorter or analyzed on a FACSymphony A5. Flow cytometry data was analyzed using FlowJo V10 software.

### Mitochondria analysis

Staining for mitochondrial membrane potential was performed as previously described in ref. ^18^. Briefly, whole BM was freshly isolated and stained with fluorochrome-conjugated antibodies for HSPC markers as described above, washed, and incubated at 37 °C and 5% CO_2_ for 30 min with TMRE (0.1 μM, Sigma). For MitoTracker staining, freshly isolated BM cells were stained with fluorochrome-conjugated antibodies for HSPC markers, incubated with 25 nM MitoTracker (ThermoFisher) in PBS supplemented with 50 μM Verapamil at 37 °C for 30 min, then washed. For MitoTracker staining, freshly isolated BM cells were stained with fluorochrome-conjugated antibodies for HSPC markers, incubated with 25 nM MitoTracker (ThermoFisher) in PBS supplemented with 50 μM Verapamil at 37 °C for 30 min, then washed. For MitoProbe^TM^ JC-1 Assay Kit (ThermoFisher), MitoProbe^TM^ Transition Pore Assay Kit (ThermoFisher), MitoProbe™ TMRM Assay Kit (ThermoFisher), Invitrogen™Rhod-2, AM, Calcium Labeling Assay Kit (ThermoFisher), and Annexin V Conjugates for Apoptosis Detection Assay Kit (ThermoFisher) were used per the manufacturer’s instructions. Stained cells were sorted using a FACSAria II or a FACSymphony S6 Sorter or analyzed on a FACSymphony A5 within 60 min of staining. Flow cytometry data was analyzed using FlowJo V10 software.

### Immunofluorescence

Sorted HSCs were resuspended in 20 μl of Stemspan SFEM II (StemCell Technologies) supplemented with 100 ng/ml rmSCF (Peprotech) and 200 ng/ml rmTPO (Peprotech) and 1% Pen-Strep (Gibco) then seeded on retronectin-coated coverslips and allowed to adhere for 2 h at 37 °C and 5% CO_2_. Cells were fixed in 4% PFA (Electron Microscopy Services) for 15 min at 4 °C and stored at 4 °C overnight. Then cells were washed once with cold PBS (Gibco) and permeablized with 0.1% Trition X-100 (Fisher Scientific) in PBS for 20 min at room temperature (RT) and blocked with 10% goat serum (Sigma Aldrich) for 20 min at RT. Rabbit polyclonal anti-TOM20 (FL-145, Santa Cruz, dilution 1:100) and DAPI containing mounting media (Vectashield, Vector Laboratories) were used for the staining. Z-stack were acquired on a Leica SP5 equipped with 63X oil immersion lens (NA 1.4), pinhole set at 1 airy unit with voxel size 80/200 nm on XY/Z. Stacks were deconvolved using the Richardson-Lucy algorithm^54^ using measured PSF. Representative image renderings were obtained by Imaris 7 (Bitplane).

### Transmission electron microscopy

For mitochondrial analysis, HSPCs (Lin-, Sca-1 + , c-Kit + ) were sorted, spun down, and red blood cells were added to assist in identifying cell pellet. Cells were fixed in 2.5% glutaraldehyde in 0.1 M sodium cacodylate buffer 0.1 M, pH 7.4 for 1 hr at RT. Samples were post-fixed in 1.0% aqueous osmium tetroxide (pH 7.4) followed by 1% uranyl acetate, dehydrated in a graded series of ethanol, and embedded in Lx112 resin (LADD Research Industries). Ultrathin (80 nm) sections were cut on a Leica UC7, contrasted with uranyl acetate followed by lead citrate, and viewed on a JEOL 1400Plus transmission electron microscope (Jeol Ltd.) at 120 kV. Images were manually segmented using ImageJ.

### Extracellular flux assays

For experiments using mouse *Dnmt3a*-mutant HSPCs, 150,000 naïve or stimulated c-Kit+ HSPCs were seeded on poly-D lysine (Sigma)-coated XF96 cell culture microplates. Oxygen consumption rate (OCR) and extracellular acidification rate (ECAR) were measured using Seahorse XF Mito Stress kit and Glycolysis Stress kits (Agilent Technologies, 102416-100) using the Seahorse XFe96 analyzer (Agilent). For experiments using *Tet2*-knockout HSPCs, c-Kit+ HSPCs were plated and cultured in Stempro plus SCF for 12 h, then transferred to microplates at 100,000 cells per well for assessment using the Seahorse XF Mito Stress kit. All tests performed on human cord blood samples were carried out using the XF96 Extracellular Flux Analyzer from Seahorse Bioscience (North Billerica, MA). The sensor cartridge was hydrated overnight in a non-CO_2_ incubator using the calibration buffer medium supplied by Seahorse Biosciences (200 μl of buffer per well) on the day prior to the assay. The wells of Seahorse XFe96 microplates were coated the following day at 4 °C with a 40 μl solution of Cell-Tak (Corning; Cat#354240) containing 22.4 μg/ml. The Cell Tak-coated Seahorse microplate wells were subsequently rinsed with distilled water. For plating, all cells were seeded at a density of 80,000 cells per well on Seahorse XFe96 microplates, using XF base minimal DMEM media containing 11 mM glucose, 2 mM glutamine, and 1 mM sodium pyruvate. Following cell seeding, 180 μl of XF base minimal DMEM medium was added to each well, and the plate was centrifuged at 100 g for 5 min. Following a one-hour incubation of the seeded cells at 37 °C in a non-CO_2_ incubator, the oxygen consumption rate (OCR) and extracellular acidification rate (ECAR) were evaluated under the baseline and in response to 1 μM Oligomycin, 1 μM carbonylcyanide-4-(trifluoromethoxy)-phenylhydrazone (FCCP), and 1 μM Antimycin and Rotenone (all from Sigma-Aldrich) using the XFe96 analyzer.

### SCENITH assay

c-Kit+ HSPCs were cultured in StemPro^TM^-34 SFM with complete nutrient supplement (Gibco) media for 1 hour at 37 °C and 5% CO_2_ in a 6-well plate. Then c-Kit+ HSPCs were transferred to a 96-well plate (150,000 cells/well) with or without inhibitors (1 μM oligomycin, Millipore Sigma; 100 mM 2-DG, Millipore Sigma; or 100 nM MitoQ, Cayman Chemical) for 1 hr at 37 °C and 5% CO_2_. Puromycin (10 μg/ml, Thermo Fisher Scientific) was then added to each well and incubated for 30 min at 37 °C and 5% CO_2_. Cells were then harvested, washed, and stained with Live/Dead viability Ghost Red 780 Dye (13-0865-T100, Tonobo) and fluorochrome-conjugated antibodies for HSPC markers as described above. Cells were then fixed and permeabilized (eBioscience Staining Buffer Set, 00-5523-00, Invitrogen) followed by intracellular staining for puromycin (clone 12D10, Alexa Fluor 647, Millipore Sigma). Cells were collected and analyzed by flow cytometry on a FACSymphony A5 (BD). Dependency and capacity were quantified using algorithms that are based on inhibiting a single metabolic pathway compared to complete inhibition of ATP synthesis^27^.

### In vitro cultures

c-Kit+ HSPCs were cultured in StemPro^TM^-34 SFM with complete nutrient supplement (Gibco) media for 16 h at 37 °C and 5% CO_2_. Addition of MitoQ (100 nM) (Cayman Chemical, 89950), IGF1 (100 ng/ml) (StemCell Technologies), m-TPP (1, 10, 100 nM) (Thermo Scientific Chemicals, AAA1587818), h-TPP (1, 10, 100 nM) (Sigma, 377538), d-TPP (1, 10, 100 nM) (Thermo Scientific Chemicals, AAA1429509), or vehicle control (DMSO) were added to the media as indicated.

### Quantitative Real-Time PCR (qRT-PCR)

HSPCs were collected after 16hrs of treatment with MitoQ or vehicle control, then washed with 1 × PBS. Whole cell DNA was extracted using the DNeasy blood and tissue kit (Qiagen, # 69504) according to the manufacturer’s protocol. The DNA was eluted in UltraPure™ DNase/RNase-free distilled water (Invitrogen, #10977015), and processed with a spectrophotometer (DeNovix®, DS-11 FX+) for concentration determination. The qRT-PCR was performed using the Power SYBR™ green PCR master mix (Applied Biosystems™, 4367659), and technical triplicates of each sample were prepared. The qRT-PCR was performed on the QuantStudio™ 7 Flex platform (Applied Biosystems™) using a standard comparative CT (ΔΔC_T_) method. The mtDNA abundance was calculated using the ΔΔC_T_ method relative to nuclear DNA.

### Single-cell multi-omics analysis of DNA Methylation and RNA expression in DNMT3A R882 mutant CD34+ cells

Single-cell DNA methylation (RRBS) data, combined with single-cell RNA sequencing (Smart-Seq2) data from CD34+ enriched cells in GCSF-mobilized bone marrow samples (*n* = 2), were retrieved from GSE158067 [GSE158067_gene_exp_mtx.txt and Supplementary Table 5 from Nam et al. ^22^]. Seurat V5.1 was used to process the scRNA data. The counts were log-normalized, scaled, and centered using the number of RNA features, mitochondrial percentage, and plates as covariates. Differential expression between the DNMT3A R882 mutant and wild-type (WT) phenotype, determined through targeted genotyping, was assessed using the Wilcoxon Rank Sum test via the limma method. Genes were ranked based on the –log10 p-value and the sign of the log fold change. To perform GSEA on the DNA methylation data, 12,131 genes were ranked by the differential methylation region (DMR) value within the ±1 kb TSS region using the –log10 *p*-value and fold change sign. The enrichment of GO:0006119 (Oxidative Phosphorylation) was evaluated using the GSEA method in ClusterProfiler 4.8 for both RNA and DNA methylation data.

### Whole Genome Bisulfite Sequencing (WGBS)

DNA was extracted from hematopoietic cells using the PureLink Genomic DNA Mini Kit (Invitrogen) and quantified using Qubit. 200 ng of genomic DNA, including 0.2% Lambda DNA (N6-methyladenine-free; NEB) was fragmented in a final volume of 50 mL using the Covaris LE220 targeting ~350 bp inserts. A 1.5x AMPure clean-up was performed post fragmentation resulting in a final volume of 20 mL. Fragmented DNA was bisulfite converted with the EZ-96 DNA Methylation-Gold Mag Prep Kit (Zymo Research) according to manufacturer recommendations. Whole genome bisulfite (WGBS) libraries were constructed with ~100 ng of bsDNA using the xGen Methyl-Seq Library Prep Kit (IDT) and unique dual indexes (IDT). Nine PCR cycles were performed during the indexing PCR step, followed by a final 0.85x AMPure cleanup. Final libraries were assessed on the bioanalyzer instrument for average library size and concentration measured by qPCR using KAPA library quantification kits (Roche). Libraries were sequenced using 2×150 paired end reads on an Illumina NovaSeq X Plus.

### Whole Genome Bisulfite Sequencing Analysis

FastqQC v0.11.8 was used to assess the quality of the raw reads. Paired end reads were trimmed to remove adapter sequences and low quality reads with Cutadapt v1.18 using the parameters (“-q 15,10 -u 10 -U 15 --minimum-length 36”) and reassessed using FastqQC. The mouse reference genome mm10 was first converted into a bisulfite converted version using Bismark v0.20.0 (bismark_genome_preparation). The paired end reads were aligned to the mm10 bisulfite converted genome using the options: “-N 1 -L 28 --score_min L,0,-0.6” and deduplicated using the deduplicate_bismark command from Bismark. Bismark command bismark_methylation_extractor was used to calculate DNA methylation levels. Bisulfite conversion was estimated using the conversion rate of cytosine to thymine in the lambda reference genome. DMRs were identified with DSS v2.43.2 using the CpG methylation with coverage and methylation counts and calling DMRs between groups using the two-group comparison for biological replicates. The DMRs were called using the following parameters: DMLtest: “smoothing = TRUE, smoothing.span = 500” and callDMR: “delta = 0.2, p.threshold = 0.001, minlen = 200, minCG = 3, dis.merge = 50, pct.sig = 0.5”. Biological replicates were combined by merging replicate -CX_report.txt files from Bismark and summing the methylated and unmethylated values per CpG locus. DMRs with missing CpG values in any of the samples were removed. Intersections for control DMR comparisons were performed and visualized by Intervene Upset function using default parameters.

### CFU assay

BM mononuclear cells were plated in MethoCult GF M3434 (StemCell Technologies) supplemented with MitoQ (100 nM), m-TPP (1 nM) (Thermo Scientific Chemicals, AAA1587818), d-TPP (1 nM) (Thermo Scientific Chemicals, AAA1429509), or vehicle and cultured at 37 °C and 5% CO_2_. Colonies were scored between 7 and 10 days post-plating. For secondary plating CFUs were harvested, counted and plated at equal cell input with supplementation. Colonies were scored using a Nikon Eclipse TS100 inverted microscope.

### MitoQ treatment

For in vivo experiments, Mitoquinol (MitoQ) or vehicle control was administered to mice by IP injection (2 mg/kg, Cayman Chemical, #89950) for 5 consecutive days.

### Targeted MitoQ Selected Reaction Monitoring (SRM) assay

Cell and mitochondrial pellets were transferred to Mass Spectrometry and Protein Chemistry Service (MSPC) at the Jackson Laboratory. Each 2 mL sample tube had a 5 mm stainless steel bead added, along with 150 µL of 2:2:1(acetonitrile: methanol: water) extraction buffer. Sample tubes were then placed in the Tissue Lyser II (QIAGEN) for 2 minutes at a frequency of 20 oscillations per second. Beads were then removed from the samples and the samples were allowed to extract for one hour at −20 °C. Sample extract was then clarified by centrifugation at 12,000 x g at 4 °C for 10 minutes. The supernatant was transferred to a new tube and the samples were dried using a vacuum centrifuge. Dried samples were then reconstituted with 20 µL of 60% acetonitrile/40% water and transferred to a sample vial for liquid chromatography tandem mass spectrometry (LC-MS/MS) SRM quantification. In addition to the individual samples, a cell lysate pool was also extracted for use as a standard eight-point quantification curve ranging from 0 pg on column to 1500 pg on column. During the LC-MS/MS analysis 2 uL equivalents of the reconstituted samples were injected onto the column per replicate (three technical replicates per sample). LC-MS/MS SRM analysis was performed on a Thermo TSQ Altis Plus mass spectrometer coupled to a Vanquish ultra high-performance liquid chromatography (UHPLC) system using a 10-minute gradient on an Agilent InfinityLab Poroshell 120 EC-C18 Column (2.1 × 50 mm 2.7 µm, part # 699775-902). Chromatography separation utilized water with 10 mM ammonium acetate and 0.1% formic acid (Buffer A) and acetonitrile with 0.1% formic acid (Buffer B). The gradient used a flow rate of 250 µL per minute and consisted of the following steps: 98% Buffer A/2% Buffer B for 1 min, ramped to 2% Buffer A/98% Buffer B by 7 minutes, held at 2% Buffer A/98% Buffer B until 8.5 minutes, and adjusted to 98% Buffer A/2% Buffer B by 9.5 minutes where it held until 10 minutes. Mito-Q (Cayman Chemical) analyte transitions (default of +1 charge) used were 585.4 m/z for the precursor ion with fragmentation ions of 288.967 m/z (collision energy = 61.98 V), 387.133 m/z (collision energy = 40.47 V), and 555.133 m/z (collision energy = 39.05 V). Other general method conditions included an autosampler temperature of 6 °C, column chamber of 40 °C, the H-ESI ion source, voltage of 3700 V, positive polarity, sheath gas of 50 (arb), auxiliary gas of 10 (arb), sweep gas of 1 (arb), an ion transfer tube temperature of 380 °C, and a vaporizer temperature of 350 °C. Specific SRM settings included a 6 second peak width, a 0.6 s cycle time, 10 points per peak, a Q1 resolution of 0.7 (FWHM), a Q3 resolution of 1.2 (FWHM), and a CID gas of 1.5 mTorr. Peak area for Mito-Q in the sample runs was then extracted using the Peak Detection in the Thermo FreeStyle 1.8 SP2 software (Version 1.8.63.0) and averages were calculated for the groups. These group peak areas were then compared to the Mito-Q standard curve of known concentrations and concentrations were calculated. Mito-Q concentrations were normalized to cell input.

### Single cell RNA-seq

c-Kit+ HSPCs were cultured with MitoQ (100 nM) or vehicle in StemPro^TM^-34 SFM with complete nutrient supplement (Gibco) for 16 h at 37 °C and 5% CO_2_. Cells were harvested and stained with fluorochrome-conjugated antibodies for HSPC markers. Lin- Sca-1+ c-Kit+ cells were sorted directly into tubes containing DMEM/10%FBS at 4 ^o^C. Cells were counted on a Countess II automated cell counter (Thermo Fisher), and 12,000 cells were loaded onto one lane of a 10X Chromium microfluidic chip (10X Genomics). Single-cell capture, barcoding, and library preparation were performed using the 10X Chromium version 3.1 chemistry according to the manufacturer’s protocol (#CG000315). cDNA and libraries were checked for quality on Agilent 4200 Tapestation and quantified by KAPA qPCR before sequencing. Libraries were sequenced using an Illumina NovaSeq 6000 S4 flow cell lane with an average sequencing depth of 75,000 reads per cell. Illumina base call files for all libraries were demultiplexed and converted to FASTQs using bcl2fastq v2.20.0.422. The Cell Ranger pipeline (10X Genomics, version 6.0.0) was used to align reads to the mouse reference GRCm38.p93 (mm10 10X Genomics reference 2020-A), deduplicate reads, call cells, and generate cell by gene digital count matrices for each library. The resultant count matrices were uploaded into PartekFlow (version 10.0.22.0428) for downstream analysis and visualization. This included log transformation of count data, principal component analysis, graph-based clustering from the top 20 principal components using the Louvain algorithm, Uniform Manifold Approximation and Projection (UMAP) visualization, and pathway enrichment analysis. Differentially expressed genes were investigated for overlap with published datasets using Gene Set Enrichment Analysis (RRID:SCR_003199).

### Human cord blood samples

Human mPB samples were obtained with informed consent from Princess Margaret Cancer Centre following the procedures approved by the University Health Network (UHN) research ethic board. Mononuclear cells were isolated by density gradient centrifugation using Ficoll-Paque ™ (Fisher Scientific, #45001749), and hematopoietic progenitors were enriched through lineage marker depletion (STEMCELL, #14056). The CD34+ cells were then isolated using the CD34 Microbead kit and purified using LS columns, following the directions provided by the manufacturer (Miltenyi Cat# 130-042-401).

### Lentivirus production and transduction

HEK293T cells were grown in RPMI 1640 medium (Wisent, #350-035-CL) supplemented with 10% FBS (Wisent, #080–150) and 1% Gluta-Plus (Wisent, #609-066-EL). Cells were seeded in 15 cm tissue culture plates (Sarstedt, #83.3903) at a density of 7 × 10^6^ cells per plate one day before transfection. On the day of transfection, cells were co-transfected with lentiviral plasmid vectors, psPAX2 (Addgene, #12259), and pCMV-VSVG (Cell biolabs, #320023) using the jetPRIME transfection reagent (Polyplus, #CA89129-924) according to manufacturer’s protocol. Supernatant containing viral particles was collected at 48- and 72- hours post transfection and filtered through a 0.45 µm PVDF membrane (Sigma, #SE1M003M00). Viral particles were precipitated in 40%(w/v) polyethylene glycol (Sigma, #89510-1KG-F) overnight. On the next day, the viral particles were collected by centrifugation at 2500 x g for 30 min at 4 °C. The pellet was resuspended in HBSS (Gibco, #14170112) + 25 mM HEPES (Thermo Fisher, #15630-080) and stored at −80 °C for long term storage. For lentiviral transductions, non-TC-treated 24 well plates were coated with 20 µg/mL of Retronectin (Takara, Cat #T100B) for 2 h at room temperature followed by aspiration and blocking with PBS containing 2% (w/v) BSA (Wisent Bioproducts, Cat #800-096-EG) for 30 min at room temperature. After aspiration of the blocking buffer, the concentrated virus suspension was added to wells. The plates were then centrifuged at 2500 x g for two hours at 4 °C to allow virus binding. Following centrifugation, unbound virus was aspirated, and cells were added. The plates were then transferred to a 37 °C incubator to initiate lentiviral infection.

### Human in vitro competition assays

Competition experiments were conducted using 96-well flat-bottom tissue culture plates (Corning, Ref #351172). Following transduction of CD34+ cells with lentiviruses encoding either shDNMT3A or shNT, cells were sorted by flow cytometry based on their fluorescent markers (BFP or GFP). DNMT3A knockdown (KD) BFP+ cells or non-targeting BFP+ control cells were subsequently mixed with non-targeting GFP+ control cells with 2000 cells per well in a proportion of 40% and 60%, respectively. The cell mixture was added to human MethoCultTM GF H4330 medium (StemCell Technologies, Cat #04330) supplemented with StemSpan™ CC100 (StemCell Technologies, Cat #02690) and BIT 9500 Serum Substitute (StemCell Technologies, Cat #09500) with a density of 2000 cells per well, treatment was administered as indicated (MitoQ at 100 nM). Cells were incubated at 37 °C with 5% CO_2_ for 14 days. Following the incubation period, cells were washed with 1X PBS, and flow cytometry was performed to determine the ratio of GFP- to BFP-positive cells. Annexin V staining was employed to exclude apoptotic cells, ensuring that the analysis was restricted to viable cell populations.

### Statistical analyses

Statistical analyses and graphing were performed using Prism 9 (GraphPad). Individual mice and/or biological replicates are presented as individual dots and/or mean ± SEM. No blinding was performed for these studies. All experiments were performed a minimum of 2 independent times. When comparing more than 2 groups, ANOVA was performed with multiple comparisons as reported in the figure legends. When only 2 groups were compared an unpaired, two-tailed Student’s *t*-test was used unless otherwise stated. Exact *p* values are reported in figures. *P*-values ≤ 0.05 were considered significant.

### Reporting summary

Further information on research design is available in the Nature Portfolio Reporting Summary linked to this article.

## Supplementary information

Supplementary Information

Reporting Summary

Transparent Peer Review file

## Source data

Source Data

## Data Availability

Raw and processed scRNA-seq data generated in this study have been deposited and are publicly available in the Gene Expression Omnibus GSE233963. Whole-genome bisulfite sequencing (WGBS) data generated in this study has been deposited and is publicly available in the Gene Expression Omnibus GSE284493. RNA-seq data from *Dnmt3a*^R878H/+^ HSCs and *Dnmt3a* KO HSCs, and WGBS data from *Dnmt3a* KO HSCs, was published by Jeong et al. ^20^ and available at the Gene Expression Omnibus GSE98191. WGBS data from *Dnmt3a*^R878H/+^ BM and *Dnmt3a* KO BM was published by Li et al. ^21^ and available at the Short Read Archive under BioProject PRJNA1008414. DNA methylation and scRNA-seq data from *DNMT3A*^R882^ CD34^+^ cells was published by Nam et al. ^22^ and is available at the Gene Expression Omnibus GSE158067. Source data are provided with this paper.
