# Supplementary material for: Elevated mitochondrial membrane potential is a therapeutic vulnerability in Dnmt3a-mutant clonal hematopoiesis
Source: Nat Commun. 2025 Apr 16;16:3306. doi: 10.1038/s41467-025-57238-2 (PMC12003737; doi:10.1038/s41467-025-57238-2)
Supplement: Supplementary file 1 — Supplementary Information [file 41467_2025_57238_MOESM1_ESM.pdf]

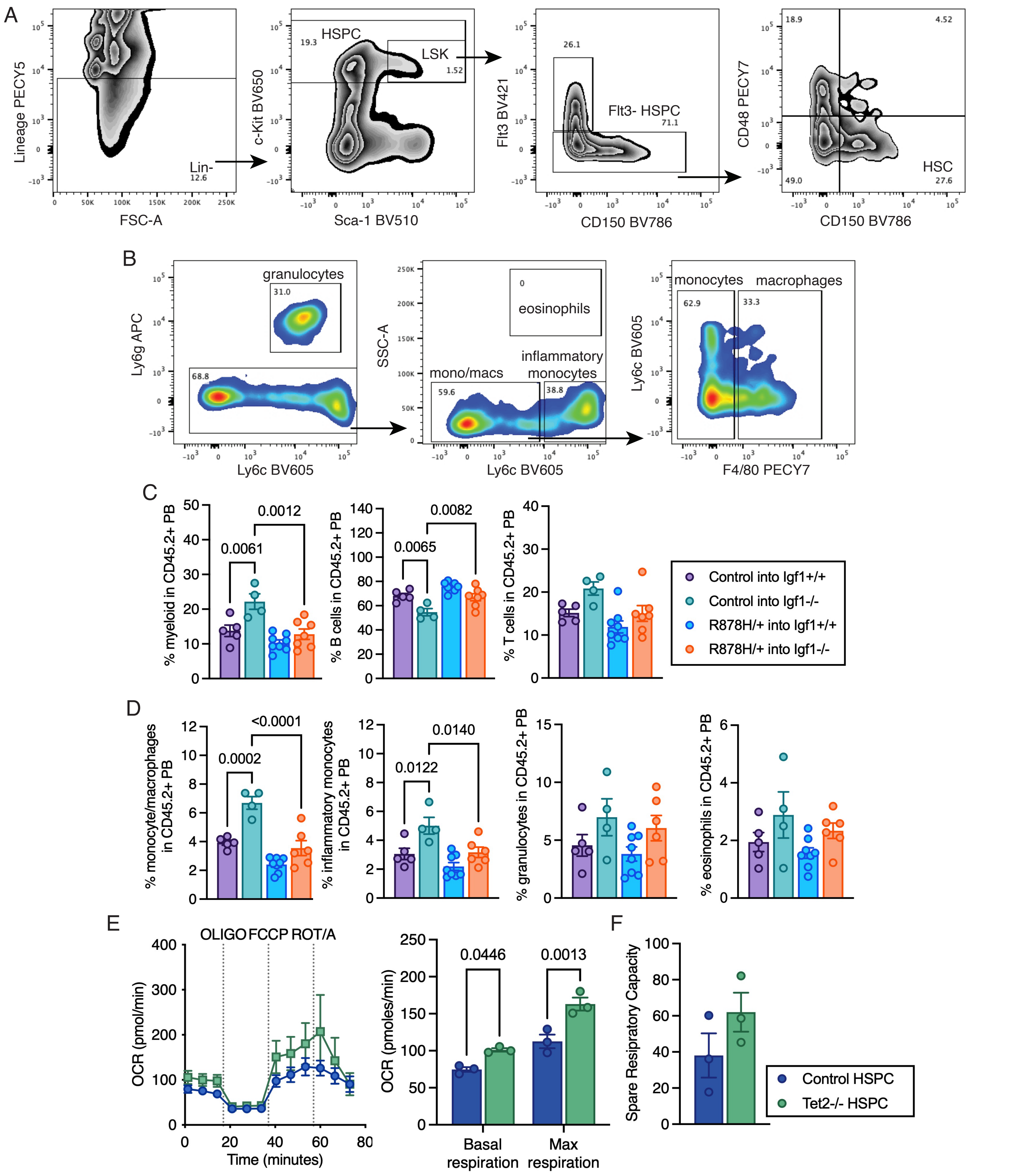
A
Lineage PECY5
FSC-A
Lin-
12.6
c-Kit BV650
Sca-1 BV510
HSPC
19.3
LSK
1.52
Flt3 BV421
CD150 BV786
26.1
Flt3- HSPC
71.1
CD48 PECY7
CD150 BV786
18.9
4.52
49.0
27.6
HSC
B
Ly6g APC
Ly6c BV605
granulocytes
31.0
68.8
SSC-A
Ly6c BV605
eosinophils
0
mono/macs
59.6
inflammatory monocytes
38.8
Ly6c BV605
F4/80 PECY7
monocytes
62.9
macrophages
33.3
C
% myeloid in CD45.2+ PB
0.0061
0.0012
% B cells in CD45.2+ PB
0.0065
0.0082
% T cells in CD45.2+ PB
Control into Igf1+/+
Control into Igf1-/-
R878H/+ into Igf1+/+
R878H/+ into Igf1-/-
D
% monocyte/macrophages in CD45.2+ PB
0.0002
<0.0001
% inflammatory monocytes in CD45.2+ PB
0.0122
0.0140
% granulocytes in CD45.2+ PB
% eosinophils in CD45.2+ PB
E
OLIGO FCCP ROT/A
OCR (pmol/min)
Time (minutes)
OCR (pmoles/min)
0.0446
0.0013
Basal respiration
Max respiration
F
Spare Respiratory Capacity
Control HSPC
Tet2-/- HSPC

Supplementary Figure 1: *Dnmt3a*$^{R878H/+}$ cells maintain lineage-balanced hematopoiesis. a, Representative gating strategy for HSPC and HSC populations. **b,** Representative gating strategy for myeloid cell populations. **c,** left to right: Frequency of myeloid cells in CD45.2+ PB. Frequency of B cells in CD45.2+ PB. Frequency of T cells in CD45.2+ PB at 28 weeks post-transplant. **d**, left to right: Frequency of monocyte/macrophage (CD11b+ Ly6G- Ly6Clo F4/80+) cells in CD45.2+ PB. Frequency of inflammatory monocytes (CD11b+ Ly6G- Ly6Chi) in CD45.2+ PB. Frequency of granulocytes in CD45.2+ PB. Frequency of eosinophils in CD45.2+ PB at 28 weeks post-transplant. **c-d**, Bars represent mean ± SEM, points from biological replicate mice (*n* = 5 control into Igf1+/+, *n* = 4 control into Igf1-/-, *n* = 8 R878H/+ into Igf1+/+, *n* = 7 R878H/+ into Igf1-/-). Statistical analyses used one-way ANOVA with Sidak's multiple comparisons test. **e**, OCR profile plot (left) used to quantify basal and maximal respiration (right) and **f**, spare respiratory capacity in control and *Tet2*$^{-/-}$ HSPCs after 12h *in vitro* culture. **e-f**, Symbols represent mean ± SEM, bars represent mean ± SEM with points from biological replicate mice (*n* = 3). Statistical analyses used two-way ANOVA with Sidak's multiple comparisons test (**e**) and one-way ANOVA with Tukey's multiple comparisons test (**f**). Source data are provided as a Source Data file.

Supplementary Figure 2

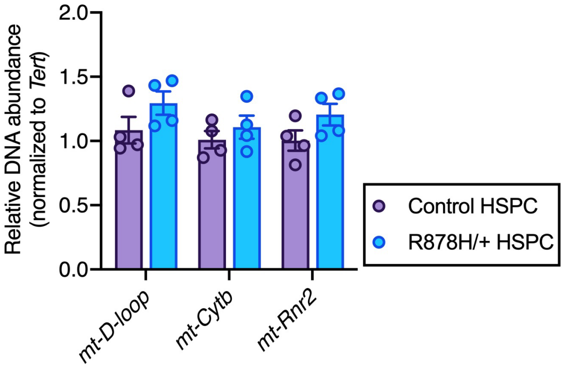

Supplementary Figure 2: Mitochondrial DNA in *Dnmt3a*$^{R878H/+}$ HSPCs. a, Quantitation of mitochondrial DNA abundance in control and *Dnmt3a*$^{R878H/+}$ HSPCs. Bars represent mean ± SEM with points from biological replicate mice (*n* = 4) biological replicate mice per genotype. Source data are provided as a Source Data file.

Supplementary Figure 3

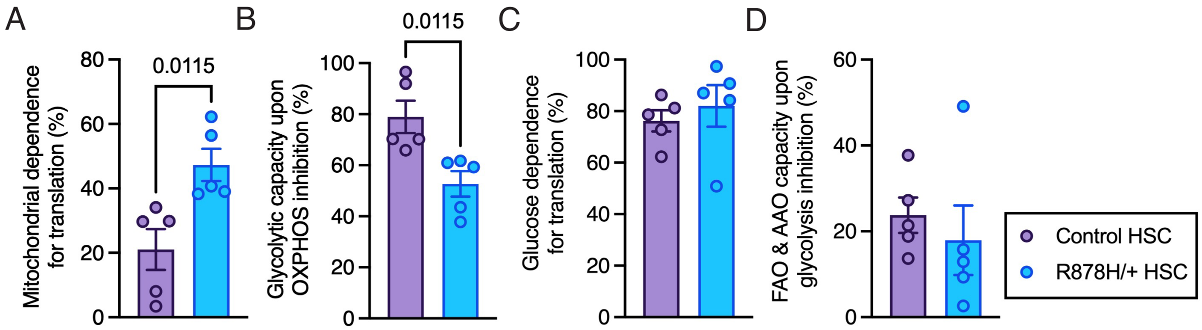

Supplementary Figure 3: Mitochondrial and glycolytic dependence and capacity in *Dnmt3a*$^{R878H/+}$ HSPCs. **a**, Mitochondrial dependence and **b**, glycolytic capacity when mitochondrial oxidative phosphorylation is inhibited. **c**, Glucose dependence and **d,** capacity to use fatty acid oxidation (FAO) and amino acid oxidation (AAO) when glycolysis is inhibited. Bars represent mean ± SEM with points from biological replicate mice (n = 5). Statistical analyses used unpaired, two-tailed t tests. Source data are provided as a Source Data file.

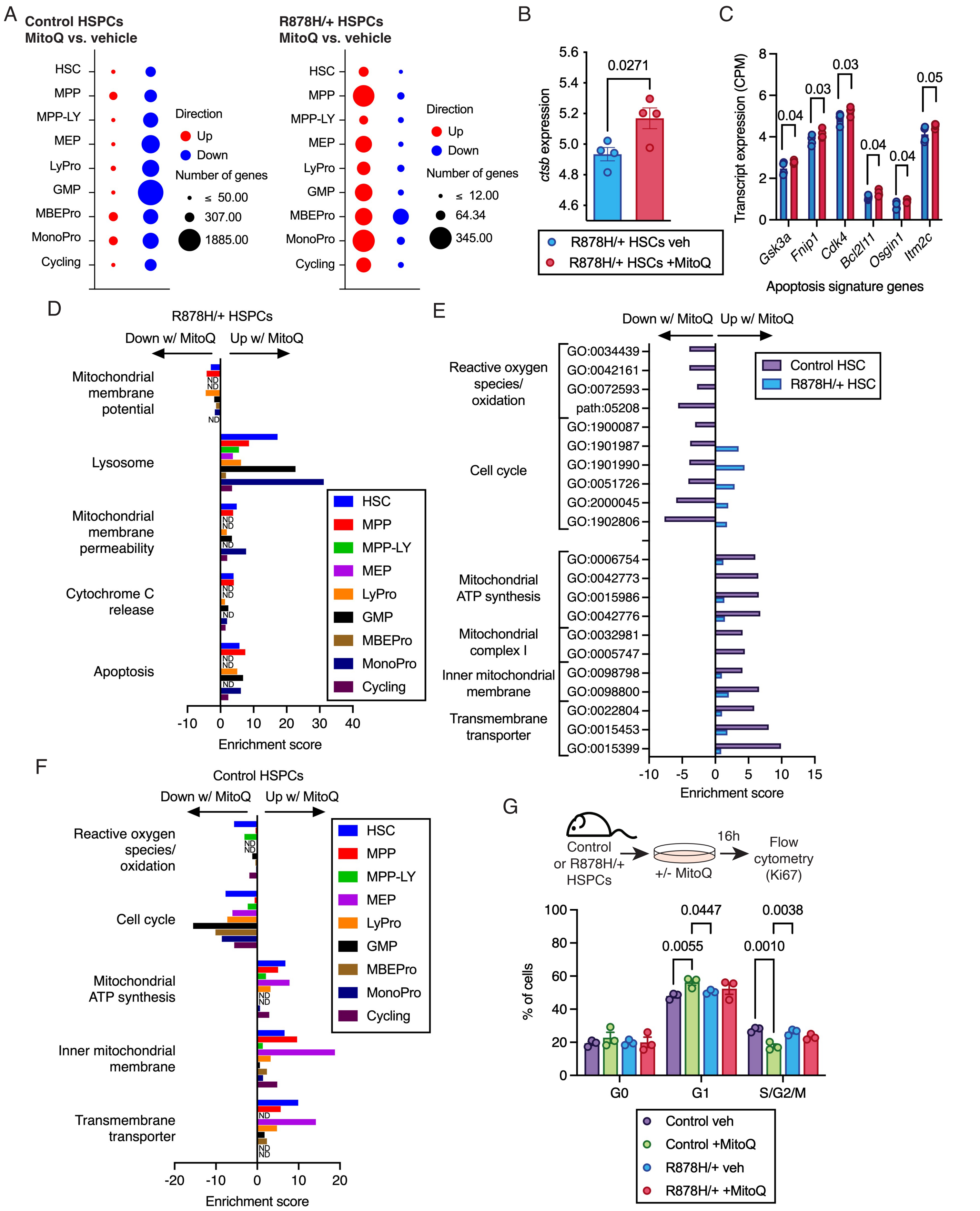
A
Control HSPCs
MitoQ vs. vehicle
R878H/+ HSPCs
MitoQ vs. vehicle
HSC
MPP
MPP-LY
MEP
LyPro
GMP
MBEPro
MonoPro
Cycling
Direction
Up
Down
Number of genes
≤ 50.00
307.00
1885.00
≤ 12.00
64.34
345.00
B
0.0271
ctsb expression
R878H/+ HSCs veh
R878H/+ HSCs +MitoQ
C
Transcript expression (CPM)
0.04
0.03
0.03
0.04
0.04
0.05
Gsk3a
Fnip1
Cdk4
Bcl2l11
Osgin1
Itm2c
Apoptosis signature genes
D
R878H/+ HSPCs
Down w/ MitoQ
Up w/ MitoQ
Mitochondrial membrane potential
Lysosome
Mitochondrial membrane permeability
Cytochrome C release
Apoptosis
ND
Enrichment score
E
Reactive oxygen species/ oxidation
GO:0034439
GO:0042161
GO:0072593
path:05208
Cell cycle
GO:1900087
GO:1901987
GO:1901990
GO:0051726
GO:2000045
GO:1902806
Mitochondrial ATP synthesis
GO:0006754
GO:0042773
GO:0015986
GO:0042776
Mitochondrial complex I
GO:0032981
GO:0005747
Inner mitochondrial membrane
GO:0098798
GO:0098800
Transmembrane transporter
GO:0022804
GO:0015453
GO:0015399
Control HSC
R878H/+ HSC
Enrichment score
F
Control HSPCs
Down w/ MitoQ
Up w/ MitoQ
Reactive oxygen species/ oxidation
Cell cycle
Mitochondrial ATP synthesis
Inner mitochondrial membrane
Transmembrane transporter
Enrichment score
G
Control or R878H/+ HSPCs
+/- MitoQ
16h
Flow cytometry (Ki67)
% of cells
0.0447
0.0038
0.0055
0.0010
G0
G1
S/G2/M
Control veh
Control +MitoQ
R878H/+ veh
R878H/+ +MitoQ

Supplementary Figure 4: *Dnmt3a*$^{R878H/+}$ and control HSC have distinct molecular responses to MitoQ treatment. a, Number of genes increased (red) and decreased (blue) in MitoQ vs. vehicle control treated Mx-Cre control HSPC populations (left) and *Dnmt3a*$^{R878H/+}$ HSPC populations (right). **b**, Transcript expression of *Ctsb* in control and *Dnmt3a*$^{R878H/+}$ HSCs treated with vehicle or MitoQ. Bars represent mean ± SEM, points from biological replicate mice (n = 4). Statistical analysis used unpaired, two-tailed t test. **c**, Transcript expression of apoptosis genes in control and *Dnmt3a*$^{R878H/+}$ HSCs treated with vehicle or MitoQ. Bars represent mean ± SEM, points from biological replicate mice (n = 4). Statistical analysis used two-way ANOVA with Fisher's LSD test. **d**, Negative and positive enrichment of gene ontology (GO) terms in MitoQ vs. vehicle treated control and *Dnmt3a*$^{R878H/+}$ HSC, MPP, MPP-LY, MEP, LyPro, GMP, MBEPro, MonoPro, Cycling populations. **e**, Negative and positive enrichment of GO terms in MitoQ vs. vehicle treated control and *Dnmt3a*$^{R878H/+}$ HSCs. **f**, Negative and positive enrichment of GO terms in MitoQ vs. vehicle treated control and *Dnmt3a*$^{R878H/+}$ HSC, MPP, MPP-LY, MEP, LyPro, GMP, MBEPro, MonoPro, Cycling populations. **g**, (top) Experimental design. (bottom) Frequency of control and *Dnmt3a*$^{R878H/+}$ HSPCs in G0, G1 and S/G2/M. Bars represent mean ± SEM, points from biological replicate mice (n = 3). Statistical analysis used two-way ANOVA with Fisher's LSD test. Source data are provided as a Source Data file.
