# Supplementary material for: Elevated mitochondrial membrane potential is a therapeutic vulnerability in Dnmt3a-mutant clonal hematopoiesis
Source: Nat Commun. 2025 Apr 16;16:3306. doi: 10.1038/s41467-025-57238-2 (PMC12003737; doi:10.1038/s41467-025-57238-2)
Supplement: Supplementary file 2 — Reporting Summary [file 41467_2025_57238_MOESM2_ESM.pdf]

Corresponding author(s): Jennifer Trowbridge

Last updated by author(s): Feb 7, 2025

Reporting Summary

Nature Portfolio wishes to improve the reproducibility of the work that we publish. This form provides structure for consistency and transparency in reporting. For further information on Nature Portfolio policies, see our Editorial Policies and the Editorial Policy Checklist.

Statistics

For all statistical analyses, confirm that the following items are present in the figure legend, table legend, main text, or Methods section.

n/a	Confirmed	
☐	☒	The exact sample size (*n*) for each experimental group/condition, given as a discrete number and unit of measurement
☐	☒	A statement on whether measurements were taken from distinct samples or whether the same sample was measured repeatedly
☐	☒	The statistical test(s) used AND whether they are one- or two-sided<br>*Only common tests should be described solely by name; describe more complex techniques in the Methods section.*
☐	☒	A description of all covariates tested
☐	☒	A description of any assumptions or corrections, such as tests of normality and adjustment for multiple comparisons
☐	☒	A full description of the statistical parameters including central tendency (e.g. means) or other basic estimates (e.g. regression coefficient) AND variation (e.g. standard deviation) or associated estimates of uncertainty (e.g. confidence intervals)
☐	☒	For null hypothesis testing, the test statistic (e.g. *F*, *t*, *r*) with confidence intervals, effect sizes, degrees of freedom and *P* value noted<br>*Give P values as exact values whenever suitable.*
☒	☐	For Bayesian analysis, information on the choice of priors and Markov chain Monte Carlo settings
☒	☐	For hierarchical and complex designs, identification of the appropriate level for tests and full reporting of outcomes
☒	☐	Estimates of effect sizes (e.g. Cohen's *d*, Pearson's *r*), indicating how they were calculated

Our web collection on statistics for biologists contains articles on many of the points above.

Software and code

Policy information about availability of computer code

Data collection	Flow cytometry data was collected using BD LSR II,FACSymphony A5, FACSAria II, and FACSymphony S6 all using FACSDiva V9.; Sequencing data was collected using NovaSeq 6000 (Illumina); qRT-PCR was performed on the QuantStudio™ 7 Flex platform (Applied Biosystems™; Imaging data was collected using Leica SP5 confocal microscope; Agilent Seahorse Wave Desktop Version 2.2.1.5 was used for the seahorse assays; TEM data was collected using a JEOL 1400Plus TEM; LC-MS/MS SRM analysis was performed on a Thermo TSQ Altis Plus mass spectrometer coupled to a Vanquish ultra high-performance liquid chromatography (UHPLC) system.
Data analysis	Flow cytometry data was processed and analyzed using FACSDiva V9 and FlowJo v9/v10; Representative image renderings were obtained by Imaris 7; TEM images were segmented using ImageJ; presentation and statistical analysis of data was performed using GraphPad Prism v9/ v10, and GSEA (v4.1.0) was used for pathway analysis,Peak area for Mito-Q in the sample runs was then extracted using the Peak Detection in the Thermo FreeStyle 1.8 SP2 software (Version 1.8.63.0)

For manuscripts utilizing custom algorithms or software that are central to the research but not yet described in published literature, software must be made available to editors and reviewers. We strongly encourage code deposition in a community repository (e.g. GitHub). See the Nature Portfolio guidelines for submitting code & software for further information.

Data

Policy information about availability of data

All manuscripts must include a data availability statement. This statement should provide the following information, where applicable:

- Accession codes, unique identifiers, or web links for publicly available datasets
- A description of any restrictions on data availability
- For clinical datasets or third party data, please ensure that the statement adheres to our policy

Raw and processed scRNA-seq data generated in this study have been deposited and are publicly available in the Gene Expression Omnibus (GSE233963). Primary WGBS sequencing data is available in the Gene Expression Omnibus (GSE284493). Source data are provided with this paper. All other data supporting the findings in this study are available from the corresponding author upon reasonable request.

Research involving human participants, their data, or biological material

Policy information about studies with human participants or human data. See also policy information about sex, gender (identity/presentation), and sexual orientation and race, ethnicity and racism.

Reporting on sex and gender	*Use the terms sex (biological attribute) and gender (shaped by social and cultural circumstances) carefully in order to avoid confusing both terms. Indicate if findings apply to only one sex or gender; describe whether sex and gender were considered in study design; whether sex and/or gender was determined based on self-reporting or assigned and methods used. Provide in the source data disaggregated sex and gender data, where this information has been collected, and if consent has been obtained for sharing of individual-level data; provide overall numbers in this Reporting Summary. Please state if this information has not been collected. Report sex- and gender-based analyses where performed, justify reasons for lack of sex- and gender-based analysis.*
Reporting on race, ethnicity, or other socially relevant groupings	*Please specify the socially constructed or socially relevant categorization variable(s) used in your manuscript and explain why they were used. Please note that such variables should not be used as proxies for other socially constructed/relevant variables (for example, race or ethnicity should not be used as a proxy for socioeconomic status). Provide clear definitions of the relevant terms used, how they were provided (by the participants/respondents, the researchers, or third parties), and the method(s) used to classify people into the different categories (e.g. self-report, census or administrative data, social media data, etc.) Please provide details about how you controlled for confounding variables in your analyses.*
Population characteristics	We used 10 umbilical cord blood samples from different consented donors from Trillium Health, Credit Valley and William Osler Hospitals.
Recruitment	The donor recruitment in this study was a completely random approach, without any specified criteria or population-based specifications.
Ethics oversight	Cord blood (CB) samples were obtained with informed consent from Trillium Health, Credit Valley and William Osler Hospitals according to procedures approved by the University Health Network (UHN) Research Ethics Board.

Note that full information on the approval of the study protocol must also be provided in the manuscript.

Field-specific reporting

Please select the one below that is the best fit for your research. If you are not sure, read the appropriate sections before making your selection.

☒ Life sciences ☐ Behavioural & social sciences ☐ Ecological, evolutionary & environmental sciences

For a reference copy of the document with all sections, see nature.com/documents/nr-reporting-summary-flat.pdf

Life sciences study design

All studies must disclose on these points even when the disclosure is negative.

Sample size	The sample sized for each experiment is indicated in the figure legend and/or the methods section. The sample size was selected to provide sufficient statistical power to identify significant differences in groups tested. No statistical methods were used to pre-determine the sample size.
Data exclusions	A small number of technical replicates were excluded from Seahorse Assays if the value(s) were highly variable and determined to be outliers. Individual mice were removed from bone marrow transplant analysis if they failed to engraft and reconstitute multilineage peripheral blood.
Replication	Data was replicated using multiple n, as well as biological repeats. Bone marrow transplants were performed with at least 4 recipients each time. All experiments were able to be reliably reproduced.
Randomization	All mice were allocated into different groups at random.

Blinding | Investigators were not blinded to the experiment.

Reporting for specific materials, systems and methods

We require information from authors about some types of materials, experimental systems and methods used in many studies. Here, indicate whether each material, system or method listed is relevant to your study. If you are not sure if a list item applies to your research, read the appropriate section before selecting a response.

Materials & experimental systems

n/a	Involved in the study
☐	☒ Antibodies
☒	☐ Eukaryotic cell lines
☒	☐ Palaeontology and archaeology
☐	☒ Animals and other organisms
☒	☐ Clinical data
☒	☐ Dual use research of concern
☒	☐ Plants

Methods

n/a	Involved in the study
☒	☐ ChIP-seq
☐	☒ Flow cytometry
☒	☐ MRI-based neuroimaging

Antibodies

Antibodies used

Flow cytometry antibodies are as follows:

Ter119 BV421 Ter119 116233 Biolegend (1:50)
B220 BUV496 RA3-6B2 612950 BD Biosciences (1:200)
CD11b APC CY7 M1/70 101226 BioLegend (1:200)
CD3e PerCP/Cy5.5 145-2C11 100328 BioLegend (1:200)
Ly6g APC 1A8 127614 Biolegend (1:200)
Ly6c BV605 HK1.4 128035 Biolegend (1:200)
F4/80 PE-Cy7 25-4801-82 Invitrogen (1:200)
CD45.1 BV650 A20 110736 BioLegend (1:50)
CD45.2 A700 104 109822 BioLegend (1:200)

Ter119 PE-Cy5 Ter119 116210 Biolegend (1:200)
CD8a PE-Cy5 53-6.7100710 Biolegend (1:200)
B220 PE-Cy5 RA3-6B2 103210 Biolegend (1:200)
Gr-1 PE-Cy5 RB6-8C5 108410 Biolegend (1:200)
CD4 PE-Cy5 RM4-5 100514 Biolegend (1:200)
CD5 PE-Cy5 53-7.3 100610 Biolegend (1:200)
CD150 BV785 TC15-12F12.2 115937 Biolegend (1:200)
cKit BV650 2B8 105853 BioLegend (1:50)
Sca-1 BV510 D7 108129 BioLegend (1:200)
Flt3 (CD135) BV421 A2F10 135313 BioLegend (1:50)
CD48 PE/CY7 HM48-1 103424 BioLegend (1:200)
CD45.2 PE 104 109808 BioLegend (1:200)
CD45.1 FITC A20 110706 BioLegend (1:200)
CD34 FITC RAM34 553733 BD Biosciences (1:100)

Antibodies for immunofluorescence: listed in methods

Validation

All antibodies used were purchased from commercial companies and have been validated by the companies. Statements for validation of antibodies can be found from Biolegend https://www.biolegend.com/en-us/quality/quality-control. Antibodies purchased from Miltenyi Biotech https://www.miltenyibiotec.com/GB-en/products/macs-antibodies/antibody-validation. For antibodies from ThermoFisher https://www.thermofisher.com/us/en/home/life-science/antibodies/invitrogen-antibody-validation.

Animals and other research organisms

Policy information about studies involving animals; ARRIVE guidelines recommended for reporting animal research, and Sex and Gender in Research

Laboratory animals

The species, strain,sex and age of animals used are described in the methods section as well as in the results/figure legends where relevant

Wild animals

N/A

Reporting on sex	Transplant experiments were done in female mice to ensure donor and recipient were sex matched. All other experiments were done with a mix of both male and female mice.
Field-collected samples	N/A
Ethics oversight	All mouse experiments and protocols were approved by The Animal Care and Use Committee at The Jackson Laboratory.

Note that full information on the approval of the study protocol must also be provided in the manuscript.

Flow Cytometry

Plots

Confirm that:

☒ The axis labels state the marker and fluorochrome used (e.g. CD4-FITC).

☒ The axis scales are clearly visible. Include numbers along axes only for bottom left plot of group (a 'group' is an analysis of identical markers).

☒ All plots are contour plots with outliers or pseudocolor plots.

☒ A numerical value for number of cells or percentage (with statistics) is provided.

Methodology

Sample preparation	Blood was collected from mice via retro-orbital sinus and red blood cells were lysed before staining. For stem and progentiro cell analysis: BM mononuclear cells were prepared from crushed femurs, tibiae, and iliac crests of an individual mouse. MNCs were isolated post 1x RBC lysis and were stained for FACs analysis. Cell populations were stained in PBS with 3% FBS.
Instrument	All BD machines used for flow: FACSAria II, LSR II, FACSSymphony A5, and FACSymphony S6
Software	Data was collected using BD FACSDiva (v9/v10) software and analyzed with FlowJo(v9/v10) software.
Cell population abundance	Purity of samples was established based on known abundance of populations, HSC are approximately .01% of the MNC BM cells of young mice and sorted cells were gated as shown in supplemental Extended Data Fig. 1 to precisely sort this population. As this is a rare population, post-sort analysis or counting was not possible.
Gating strategy	All cells were gated on size (FSC-A/SSC-A), single cell gates (SSC-H/SSC--W and FSC-H/FSC-W) and viable (DAPI-).

☒ Tick this box to confirm that a figure exemplifying the gating strategy is provided in the Supplementary Information.
